# Human microRNA miR-197-3p positively regulates HIV-1 virion infectivity through its target DDX52 by stabilizing Vif protein expression

**DOI:** 10.1016/j.jbc.2025.108198

**Published:** 2025-01-16

**Authors:** Anindita Dasgupta, Anjali Tripathi, Alapani Mitra, Payel Ghosh, Manas Kumar Santra, Debashis Mitra

**Affiliations:** 1 National Centre for Cell Science, SP Pune University Campus, Pune, Maharashtra, India; 2 Bioinformatics Centre, SP Pune University, Pune, Maharashtra, India

**Keywords:** HIV, microRNA, RNA helicase, protein degradation, ubiquitination, host-pathogen interaction

## Abstract

MicroRNAs are a part of the integral regulatory mechanisms found in eukaryotic cells that help in maintaining cellular homeostasis by modulating the expression of target genes. However, during stress conditions like viral infection, the expression profile of the microRNAs change, thereby directly modulating the expression of viral genes and/or indirectly targeting the virus by regulating the host genes. The present study intends to identify previously uncharacterized cellular microRNAs, which are significantly modulated upon HIV-1 infection. With the available microarray data of five independent studies in the NCBI GEO database, 10 common yet functionally uncharacterized microRNAs that are deregulated during HIV-1 infection in humans were identified. Their expression profiles were validated in HIV-1 infected human peripheral blood mononuclear cells and a CD4^+^T cell line. Among them, miR-197-3p showed significant upregulation during HIV-1 infection in all the cell types tested and was selected for further characterization. We then found that miR-197-3p increases progeny virion infectivity through restricting the expression of DDX52. Interestingly, DDX52 showed a negative impact on virion infectivity by downregulating the HIV-1 viral infectivity factor (Vif) at the protein level. Mechanistically, our study also revealed that Vif, DDX52, and APOBEC3G form a complex, which might be responsible for Vif downregulation by proteasomal degradation. Taken together, our results demonstrate that miR-197-3p is a positive regulator of HIV-1 infectivity as it enhances the progeny virion infectivity by targeting DDX52, which is a negative regulator of Vif.

The hallmark of HIV infection is the systemic depletion of the CD4^+^ T cells, followed by progressive impairment in cellular immunity. This leads to the ultimate condition called AIDS, where the host becomes home to several opportunistic infections and cancers that can ultimately lead to death.

MicroRNAs are host factors that fall under the category of small noncoding RNAs and are about 19 to 21 nucleotides in length. More than 2000 human microRNAs have been discovered till date, which collectively orchestrate the expression and function of nearly one-third of all human genes (1, 2). They regulate important biological functions like cell growth and differentiation, apoptosis, killing of pathogens, and so on (3, 4). They have specific “seed sequences” by which they target mRNAs and thus prevent protein synthesis. This is achieved either by degradation or inhibition of translation process of target mRNAs (5). The last 10 years have seen tremendous growth in the area of microRNA research in various fields including pathogen-associated diseases. Hence, altered expression of many microRNAs and their functional implications have been reported upon HIV-1 infection also (6). The changes in the microRNA profiles have been detected by various advanced methods like quantitative real-time PCR (qRT-PCR), microarray, and small RNA sequencing (7, 8, 9). For instance, Triboulet *et al.* showed for the first time that the RNAi machinery indeed plays a crucial role in HIV-1 infection as the knockdown of the two RNases, Dicer and Drosha, increased the expression of HIV in the infected cells (10). The pioneering report showing the relationship between HIV-1 infection and microRNA expression came from the study of Yeung *et al.* (11). The study involved a novel technique called RNA-primed array-based Klenow enzyme (RAKE) microRNA microarray, which compared the expression of microRNAs in pNL4.3 transfected and untransfected cells. Following this, efforts were made to understand how HIV-1 modulates microRNAs and *vice versa* during HIV-1 infection. It was reported by two independent groups that microRNAs like 29a, 29b, 29c, 26a, and 21 were downregulated in HIV-1 seropositive patients (12, 13). In a comparison between resting and activated CD4^+^T cells, it was found that microRNAs 125b, 382, 150, 28, and 233 were highly upregulated in the resting T cells (14). Also, another study suggested that a pool of antagomirs (a class of chemically synthesized oligonucleotides designed to silence endogenous microRNAs) could act to reverse HIV-1 latency (15). This suggests the role of microRNAs in promoting latency by suppressing the translation of HIV-1-encoded proteins. Such evidences led to the agreement that specific microRNA signatures exist upon HIV-1 infection. For example, Ahluwalia *et al.* reported that miR-29a has a binding site on HIV-1 *nef* gene, thereby reducing its expression and consequently the viral load (16). Subsequently, many reports demonstrated that microRNAs could regulate many viral genes (17, 18). Some of the examples include miR-133b and 138 that target *env*, miR-326 targets *5′ LTR*, miR-92a targets *pol*, and *gag* is targeted by miR-149 (17). During HIV-1 infection, microRNAs also target host genes to modulate the infection. For example, miR-198 targets Cyclin T1 (which is crucial for viral gene transcription) in the monocytes to restrict the virus (18). Also, in recent times, miR-155 has emerged as a biomarker in activated T cells of HIV-infected individuals on antiretroviral therapy, where the levels of this microRNA were found to be elevated (19, 20). Hence, from the aforementioned literature, it can be inferred that microRNAs are involved in HIV-1 infection as pro- or anti-HIV factors. However, it would be of great interest to do a comprehensive analysis to identify common yet significantly deregulated microRNAs across different studies involving HIV-1 infected individuals.

The life cycle of HIV shows that it uses the host machinery, RNA metabolism being one of them, to establish a successful infection. As HIV does not possess any RNA helicase of its own, it solely depends on the host RNA helicases for its reverse transcription, transcription of the HIV mRNA, and transport of this viral mRNA from the nucleus to the cytoplasm (21). Roy *et al.* in 2006 proved this by showing how HIV-1 Gag associates with RNA helicase A to facilitate virion packaging (22). DDX3, among others was also found to be positively involved in various stages of the virus life cycle like mRNA export, translation of the newly formed viral RNAs, and budding (23, 24). On the other hand, studies have shown that overexpression of another RNA helicase Mov10, which is also a P-body marker protein, leads to decreased steady-state levels of HIV-1 Gag protein. Mov10 reduces Gag processing and enhances production of less infectious progeny virion particles by getting incorporated into them (25). Thus, it can be inferred that RNA helicases possess the capability of both facilitating and impeding HIV-1 infection.

There are several restriction factors identified in the host that are potent antiviral agents as they can restrict HIV-1 replication and infectivity. The apolipoprotein B mRNA-editing enzyme, catalytic polypeptide (APOBEC) family of proteins (A3A to A3H) is one of them that are known to interfere with the life cycle of the virus at several stages (26). As a counter defense, the virus encodes its accessory proteins that neutralize the actions of these restriction factors (27). For example, viral infectivity factor (Vif) antagonizes the actions of APOBEC3G (A3G) by causing the latter’s proteasomal degradation. In contrast, A3G restricts the viral replication by causing hypermutations in the viral genome (28) and proteasomal degradation of Vif (29). Hence, a constant conflict between the two opponents decides the fate of infection in the host cells.

In the present work, we have tried to identify commonly expressed and significantly modulated microRNAs in HIV-1 infected human samples from microarray datasets available in the publicly accessible NCBI Gene Expression Omnibus (GEO) database. miR-197-3p emerged as one of the significantly upregulated microRNAs during HIV-1 infection in all the datasets, which is still uncharacterized in the field of HIV research. Our study revealed that miR-197-3p has positive influence on the progeny virion infectivity during HIV-1 infection. Interestingly, DDX52, a target of miR-197-3p, showed a negative impact on virion infectivity during HIV-1 infection. Subsequent analysis revealed that DDX52 has a regulatory effect on HIV-1 Vif protein *via* its association with A3G during HIV-1 infection. Collectively, our study establishes a connection between two crucial host factors, microRNAs and RNA helicases, shedding new light on the interplay between host and pathogen during HIV-1 infection.

## Results

### *In silico* analysis of differentially expressed microRNA datasets available from HIV-1 infected individuals

**Figure 1:**
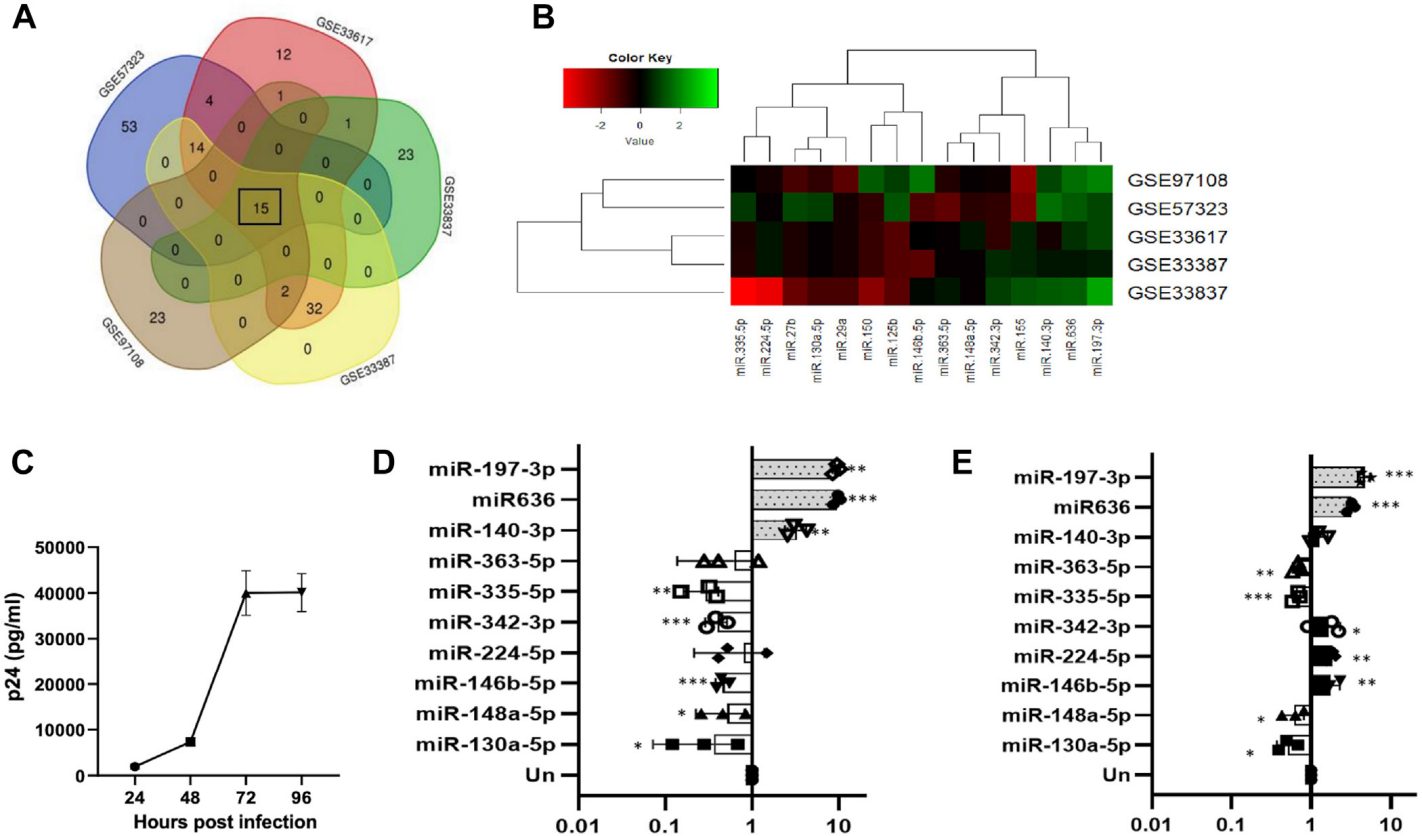
**Identification of differentially expressed microRNAs from the selected microarray datasets in NCBI-GEO database and validation of the identified and selected microRNAs in HIV-1 infected human peripheral blood mononuclear cells.***A*, Venn diagram showing the number of differentially expressed microRNAs in the five datasets, as analyzed *in silico*. The data sets used for analysis are GSE57323, GSE33387, GSE33617, GSE33837, and GSE97108. *B*, heatmap and cluster analysis of microRNA expression in HIV-1 infected human samples. The expression of differentially expressed common 15 microRNAs (indicated by the *columns*) across all the five datasets (indicated by the *rows*) are represented in the heat map. Differentially upregulated and downregulated microRNAs are represented by *green* (Fold change > 2) and *red* (Fold change < - 2) colors, respectively. *C*, successful progression of HIV-1 infection in hPBMCs as analyzed by p24 antigen capture ELISA. hPBMCs were infected with 0.5 MOI HIV-1 NL4.3 virus. The cell culture supernatants were harvested every 24 h for p24 ELISA. *D*, validation of selected 10 microRNAs identified as significantly modulated by bioinformatic analysis in HIV-1 infected hPBMCs. The graph represents fold change in the expression of the selected microRNAs at the peak of HIV-1 infection (0.5 MOI) in hPBMCs (72 h post infection), as analyzed through quantitative Real-Time PCR. U6 snRNA served as the internal control. *E*, validation of differential expression of selected 10 microRNAs in HIV-1 infected Jurkat J6 cells. Among the 10 microRNAs, six showed similar pattern whereas four (marked in *black*) did not show a similar pattern of expression during HIV-1 infection as observed in the bioinformatic analysis. Jurkat J6 cells were infected as described in (*D*) and the expression of the microRNAs were analyzed by quantitative real-time PCR. U6 served as the internal control. The results represent mean ± S.D. from n = 3 independent experiments analyzed by one-way ANOVA with Dunnett’s *post hoc* test. Statistical significance is defined as ns = *p* ≥ 0.05, ∗ = *p* ≤ 0.05, ∗∗ = *p* ≤ 0.01, ∗∗∗ = *p* ≤ 0.001. GEO, Gene Expression Omnibus; MOI, multiplicity of infection

For the present study, we selected five high-throughput microRNA microarray datasets from the NCBI GEO (https://www.ncbi.nlm.nih.gov/geo/). The GSE IDS along with PMIDs, year, platform, and the number of HIV-1 infected samples and uninfected controls are shown in Table 1. These studies obtained data from HIV-1 infected individuals across diverse geographic regions, with no prior record of any coinfection or any antiretroviral therapy. Noticeably, we have exclusively focused on human sample data and excluded the microarray datasets derived from HIV-1 infected or transfected cell lines, to enhance the physiological relevance of our analysis. Next, we used these five datasets to compare the differential expression of microRNAs between uninfected (controls) and HIV-1 infected (tests) samples with the help of GEO2R interactive web tool (as mentioned in the Experimental procedures section). False discovery rates (FDRs) or false positives are issues in identifying the correct set of differentially expressed genes or microRNAs (DEMs) when working with array-based datasets. To assess the FDR, Benjamini and Hochberg method was applied to correct for multiple comparisons and adjusted-p values were selected for identifying the correct set of DEMs. Based on the logFC and adj.*p* value cutoff criteria, around 200 microRNAs showed significant alteration of expression upon HIV-1 infection. The result of the comparative microRNA data analysis of the selected datasets is presented in the form of a Venn diagram (Fig. 1*A*). Among them, 15 microRNAs were found to be common in all the five studies and are listed in Table 2. It was found that five of the 15 microRNAs namely, miR-125b, 155, 150, 27b, and 29a have already been implicated and functionally characterized in HIV-1 infection (14, 15, 16, 17), thus confirming the validity of the current analysis. The differential expression of these 15 microRNAs is also represented using a heat map plot across the five datasets as shown in Fig. 1*B*. The cluster analysis integrated with the heat map representation was used to put microRNAs with similar expression patterns in the same group. For instance, miR-140-3p, mir-636, and miR-197-3p showing upregulation in HIV-1 infected samples have been grouped together. Similarly, two microRNAs namely, miR-29a and miR-224-5p which were downregulated across all the experiments have been clustered together. This observation suggests toward a possible involvement of these microRNAs in driving important regulatory processes during HIV-1 infection. However, expression pattern of other microRNAs like miR-342-3p and miR-335-5p could not be clearly defined on the basis of *in silico* analysis. When we looked into the literature for a possible explanation, we came across such reports which states that microRNA expression patterns may vary from person to person with respect to age, gender, immune status, geographical location, and so on (30, 31).

**Table 1:** Microarray datasets from GEO database used in the present study for analysis of differential expression of microRNAs during HIV-1 infection in humans

Accession ID	PMID (Ref. No.)	Platform	Infected samples	Uninfected (control) samples
GSE57323	25225963 (69)	GPL17391 (Applied Biosystems Human MicroRNA Array v2.0)	n = 8	n = 5
GSE33387	22240256 (70)	GPL14822 (NanoString n Counter Human miRNA assay)	n = 6	n = 8
GSE33617	22240256 (70)	GPL14842 (Applied Biosystems Human Taqman MicroRNA Array v3.0)	n = 6	n = 6
GSE33837	21829495 (71)	GPL14907 (SA Biosciences Human Genome V2.0 miRNA PCR Arrays:MAH-3200)	n = 6	n = 6
GSE97108	Yet to publish	GPL23231 (Applied Biosystems Taqman Low Density Array Human microRNA card A + B v3.0)	n = 4	n = 4

**Table 2:** List of significantly modulated microRNAs which are found to be common in all the microarray datasets listed in Table 1

microRNA modulation	Count	Names^a^
Upregulated microRNAs	4	hsa-miR-155^a^, hsa-miR-140-3p, hsa-miR-197-3p, hsa-miR-636
Downregulated microRNAs	11	hsa-miR-125b^a^, hsa-miR-150^a^, hsa-miR-27b^a^, hsa-miR-29a^a^, hsa-miR-130a-5p, hsa-miR-148a-5p, hsa-miR-146b-5p, hsa-miR-224-5p, hsa-miR-342-3p, hsa-miR-335-5p, hsa-miR-363-5p

a Functionally characterized in HIV-1 infection.

### *In-cellulo* validation of selected microRNAs identified from the *in silico* analysis

We then validated the expression profile of the selected uncharacterized 10 microRNAs from the list in Table 2 by qRT-PCR technique. For this, human peripheral blood mononuclear cells (PBMCs) from three anonymous donors of blood bank were infected with 0.5 multiplicity of infection (MOI) of HIV-1 NL4.3 virus. Fig. 1*C* shows successful progression of infection with peak of infection observed at 72 h post infection. Therefore, we analyzed microRNA expression profile of the abovementioned 10 microRNAs at 72 h following infection in human peripheral blood mononuclear cells (hPBMCs). Among them, three microRNAs including miR-197-3p, miR-140-3p, and miR-636 were found to be upregulated and the rest were downregulated upon HIV-1 infection (Fig. 1*D*), which was consistent with the results obtained from microarray datasets. Majority of the selected microRNAs also displayed a similar expression profile in a HIV-1 infected CD4^+^T cell line, Jurkat J6 (Fig. 1*E*). Among the significantly upregulated microRNAs, miR-197-3p showed a similar expression pattern in both infected hPBMCs and Jurkat J6 cells. Though this microRNA has been reported to have functional implications in some cancers (32, 33), however, its role during HIV-1 infection is yet to be studied. Hence, we selected miR-197-3p as our candidate microRNA for functional characterization during HIV-1 infection in the present study.

### miR-197-3p positively regulates progeny virion infectivity

**Figure 2:**
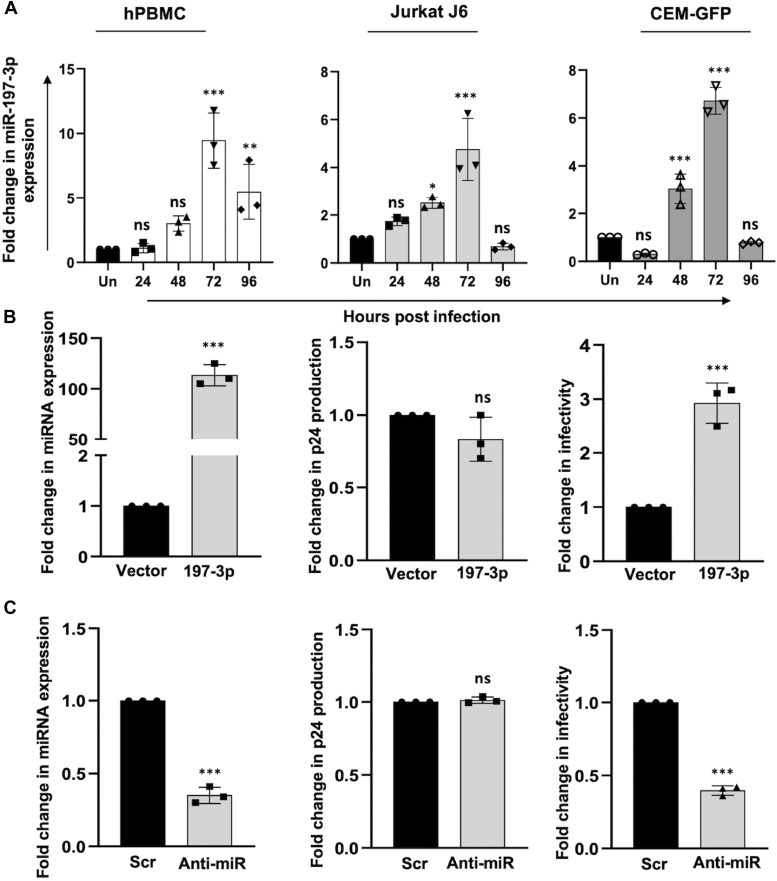
**miR-197-3p expression is increased during HIV-1 infection progression and positively regulates progeny virion infectivity.***A*, miR-197-3p expression gradually increases with HIV-1 infection progression and tends to decrease after the peak of infection. Activated hPBMCs and two CD4+T cell lines (Jurkat J6 and CEM-GFP) were infected with HIV-1 (0.5 MOI) and cells were harvested everyday till day 4. RNA was extracted followed by expression analysis by quantitative real-time PCR. U6 was used as internal control. *Un*, uninfected. The data were analyzed by one-way ANOVA with Dunnett’s *post hoc* test. *B*, mir-197-3p overexpression does not change virus production but increases the infectivity of the progeny virion particles. miR-197-3p was overexpressed in Jurkat J6 cells followed by HIV-1 infection at 0.5 MOI 24 h post transfection. The culture supernatants were used for determining virus production by p24 ELISA 48 hpi and for the comparison of progeny virion infectivity by X-gal staining in TZM-bl reporter cells. The data were analyzed by two-tailed unpaired Student’s *t* test. *C*, inhibition of miR-197-3p by anti-miR shows decreased progeny virion infectivity, without any alteration in virus production. Jurkat J6 cells were transfected with miR-197-3p inhibitor followed by HIV-1 infection (0.5 MOI) 24 h post transfection. As described in B, the culture supernatants were used for p24 ELISA and TZM-bl *X*-gal staining assay. The data were analyzed by two-tailed unpaired Student’s *t* test. The results represent mean ± S.D. from n = 3 independent experiments and statistical significance is defined as ns = *p* ≥ 0.05, ∗ = *p* ≤ 0.05, ∗∗ = *p* ≤ 0.01, ∗∗∗ = *p* ≤ 0.001. MOI, multiplicity of infection; X-gal, 5-bromo-4-chloro-3-indolyl-β-D-galactopyranoside.

Thus far, we have shown that the expression of miR-197-3p increases in HIV-1 infected cells at peak of infection which was also suggested by our microarray data analysis of all the five datasets available in the public domain. We then analyzed the expression profile of miR-197-3p through the course of infection. Human PBMCs and CD4^+^ T cell lines Jurkat J6 and CEM-GFP were infected with 0.5 MOI of the HIV-1 NL4.3 virus. It was observed that the expression of miR-197-3p increases initially with infection progression and reaches its maximum level at the peak of infection (72 h), after which it goes down significantly (Fig. 2*A*). We then wanted to see the functional implication of upregulation and knockdown of miR-197-3p on the virus. Results of the p24 antigen capture ELISA of the supernatants from both overexpression and knockdown of miR-197-3p in Jurkat J6 cells showed no significant change in the virus production (Fig. 2, *B* and *C* middle panel). However, the infectivity assay in the TZM-bl indicator cells showed that with overexpression of the microRNA (Fig. 2*B* left panel), there was an increase in progeny virion infectivity (Fig. 2*B* right panel), while microRNA knockdown (Fig. 2*C* left panel) showed a decrease in progeny virion infectivity (Fig. 2*C* right panel). Thus, our results suggest that miR-197-3p positively regulates HIV-1 infectivity.

### Identification of miR-197-3p target genes by *in silico* analysis and validation of selected targets *in cellulo*

**Figure 3:**
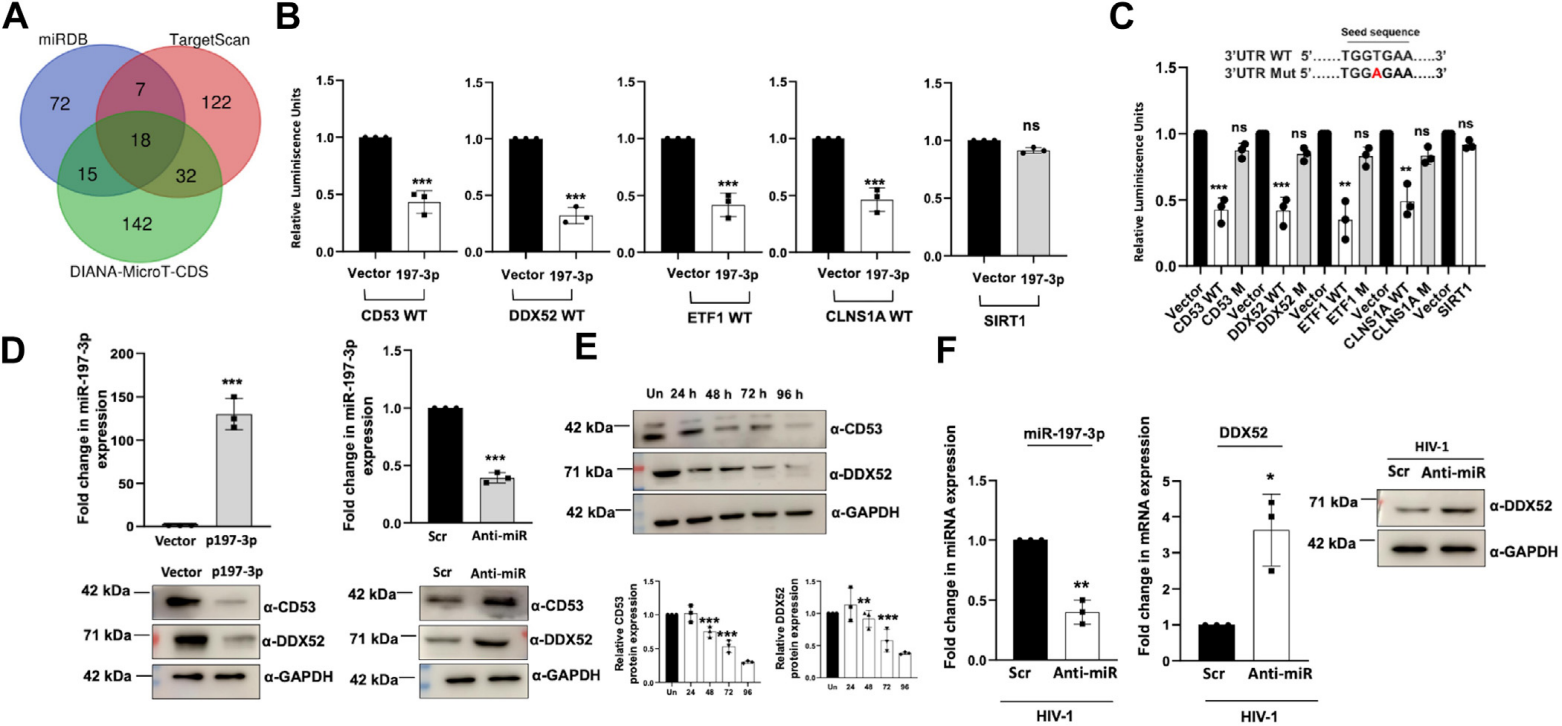
**Validation of miR-197-3p target genes identified by *in silico* analysis and downregulation of the selected target DDX52 during HIV-1 infection.***A*, Venn diagram showing the common targets of miR-197-3p obtained from the three databases namely, TargetScan, DIANA-MicroT-CDS, and miRDB. *B*, miR-197-3p suppresses the expression of its target genes as analyzed by reporter-based luciferase assay. pMIR-REPORT vectors containing the 3′UTRs of CD53, DDX52, ETF1, CLNS1A, and SIRT1 (negative control), were cotransfected with either empty vector or p-miR-197-3p along with pEGFPN1 in HEK-293T cells and harvested after 48 h for luciferase assay, and the results were normalized to GFP readings. The data were analyzed by two-tailed unpaired Student’s *t* test. *C*, mutations in the seed sequences of the target genes lead to loss of reporter activity suppression in presence of miR-197-3p. HEK-293T cells cotransfected either with WT or mutated 3′UTR seed sequences along with p-miR-197-3p were harvested after 48 h for luciferase assay and the results were normalized to GFP readings. The seed sequence with the single nucleotide mutation in the mutants is depicted above the bar graph. *WT*; *M*, mutant. The data were analyzed by one-way ANOVA with Dunnett’s *post hoc* test. *D*, miR-197-3p expression inversely correlates with the expression of its targets. Normal Jurkat J6 cells were transfected with p197-3p (*left* panel) and anti-miR-197-3p (*right* panel). The cells were harvested after 48 h and analyzed by quantitative real-time PCR and immunoblotting for CD53 and DDX52. The data were analyzed by two-tailed unpaired Student’s *t* test. *E*, CD53 and DDX52 protein expression is downregulated during HIV-1 infection in Jurkat J6 cells. Jurkat J6 cells were infected with HIV-1 (0.5 MOI) and were harvested every 24 h till day 4 for expression analysis by immunoblotting. GAPDH was used as internal control. *F*, inhibition of miR-197-3p during HIV-1 infection leads to increased expression of DDX52. Jurkat J6 cells were infected with HIV-1 followed by transfection of anti-miR-197-3p. Cells were harvested at the peak of infection (72 h) for expression analysis by quantitative real-time PCR and immunoblotting. The data were analyzed by two-tailed unpaired Student’s *t* test. The results represent mean ± S.D. from n = 3 independent experiments and statistical significance is defined as ns = *p* ≥ 0.05, ∗ = *p* ≤ 0.05, ∗∗ = *p* ≤ 0.01, ∗∗∗ = *p* ≤ 0.001. MOI, multiplicity of infection; ETF1, eukaryotic translation termination factor 1.

The gene silencing pathway shows that microRNAs work by binding to the seed sequences of their targets, thereby lowering the expression of those genes. Using online tools like TargetScan, DIANA-MicroT-CDS, and miRDB, we have identified the targets of miR-197-3p. The cutoffs applied for the selection of the targets were, −0.30 for TargetScan, 0.90 for DIANA- MicroT CDS, and 70 for miRDB. The Venn diagram presented as Fig. 3*A* gives us an idea of the number of common top scoring targets that are identified by all the three online tools. The common targets obtained from all three datasets are listed in Table 3 from the highest to lowest scores. We selected the top four best-scoring genes CD53, DDX52 (DExD-box helicase 52), ETF1 (eukaryotic translation termination factor 1), and CLNS1A (chloride nucleotide-sensitive channel 1A) for validation. Although some of the targets after CLNS1A have good scores like CTNND1 and SERTAD4, we have excluded them from our present study as they did not show significant expression in CD4+ T cells when tested *via* qRT-PCR (data not shown). We then validated the abovementioned four genes as the targets of miR-197-3p. For that, the 3′UTRs of these four genes were cloned in the pMIR-REPORT reporter vector as described in Experimental procedures. The 3′UTR of the SIRT1 gene was chosen as the negative control as it is not a predicted target of miR-197-3p. Cotransfection of miR-197-3p and 3′UTR reporter clones of these target genes in HEK-293T cells demonstrated a reduced luciferase reading as compared to the control, indicating the suppression of targets by the microRNA (Fig. 3*B*). To further confirm this finding, we determined the “seed sequences” or the sequences where the microRNA binds to bring about the suppression of its targets, through the target prediction tools. Next, we made single-nucleotide mutations in those sequences of the 3′UTR by site-directed mutagenesis (Fig. 3*C* upper). As expected, the reporter-based luciferase assay showed almost zero inhibition with the mutated reporter clones as compared to the wild type (WT) ones (Fig. 3*C* lower). Next, literature mining was done in order to determine any functional implication of these four validated target genes during HIV-1 infection. *In silico* prediction tools identified them as host proteins whose expressions are modulated during HIV-1 infection (34, 35). We then decided to look at the impact of miR-197-3p expression on the two top-scored target genes, CD53 and DDX52. Both overexpression and knockdown of the microRNA revealed its inverse correlation with CD53 and DDX52 expression, thus validating them again as the targets of miR-197-3p (Fig. 3*D*). Time kinetic expression profile of both CD53 and DDX52 in 0.5 MOI HIV-1 NL4.3 infected Jurkat J6 cells revealed a decrease in protein expression with infection progression (Fig. 3*E*), which is inversely correlated with the expression pattern of its regulatory microRNA, miR-197-3p (Fig. 2*A*). Although CD53 showed the highest score and was validated as a target of miR-197-3p, we did not select it for our study as its role in HIV-1 infection is already well characterized (36). Moreover, the second best target was DDX52, a DEAD box RNA helicase, whose function is uncharacterized with respect to HIV-1 infection. Studies have shown that many RNA helicases play a major role during HIV-1 infection, as the virus lacks its own helicase. It has also been reported that RNA viruses have the capacity to hijack the host RNA helicases to maintain their survival in the host (37). Additionally, RNA helicases are crucial for RNA processing in eukaryotes and are also vital host factors that maintain the internal homeostasis. Based on all this information, we decided to select DDX52 for further characterization in the present study. In order to verify that the downregulation of DDX52 during HIV-1 infection is caused by the elevated expression of the microRNA, we analyzed the expression of DDX52 by silencing the microRNA during HIV-1 infection. Our results demonstrated that with knockdown of miR-197-3p, the expression of DDX52 increased at the peak of infection, both at mRNA and protein levels (Fig. 3*F*). These findings clearly suggest that miR-197-3p downregulates its target DDX52 during HIV-1 infection.

**Table 3:** List of targets of miR-197-3p obtained from the three target prediction databases—TargetScan Human8.0, DIANA tools and miRDB

Genes	GenBank accession	TargetScan (total context score)	DIANA (miTG score)	miRDB (target score)
CD53	NM_001320638	−0.91	0.95	92
DDX52	NM_007010.5	−0.35	0.94	97
ETF1	NM_001291975	−0.59	0.94	95
CLNS1A	NM_001293	−0.49	0.93	91
CTNND1	NM_001085458	−0.36	0.99	93
SERTAD4	NM_019605	−0.65	0.93	95
PTPN9	NM_002833	−0.66	0.99	97
CILP	NM_003613	−0.32	0.97	97
IGFBP3	NM_000598	−0.54	0.96	92
ATP6V1A	NM_001690	−0.40	0.97	88
ARMC1	NM_001286702	−0.31	0.96	89
HNRNPD	NM_031369	−0.44	0.91	87
SYNGR1	NM_004711	−0.45	0.99	79
HEATR5B	NM_019024	−0.35	0.94	82
PPP1CC	NM_001244974	−0.37	0.98	74
NLK	NM_016231	−0.37	0.99	73
AFAP1L2	NM_001287824	−0.30	0.93	73
CHIC2	NM_012110	−0.32	0.93	70

### DDX52 negatively regulates progeny virion infectivity by decreasing the cellular expression of HIV-1 Vif

**Figure 4:**
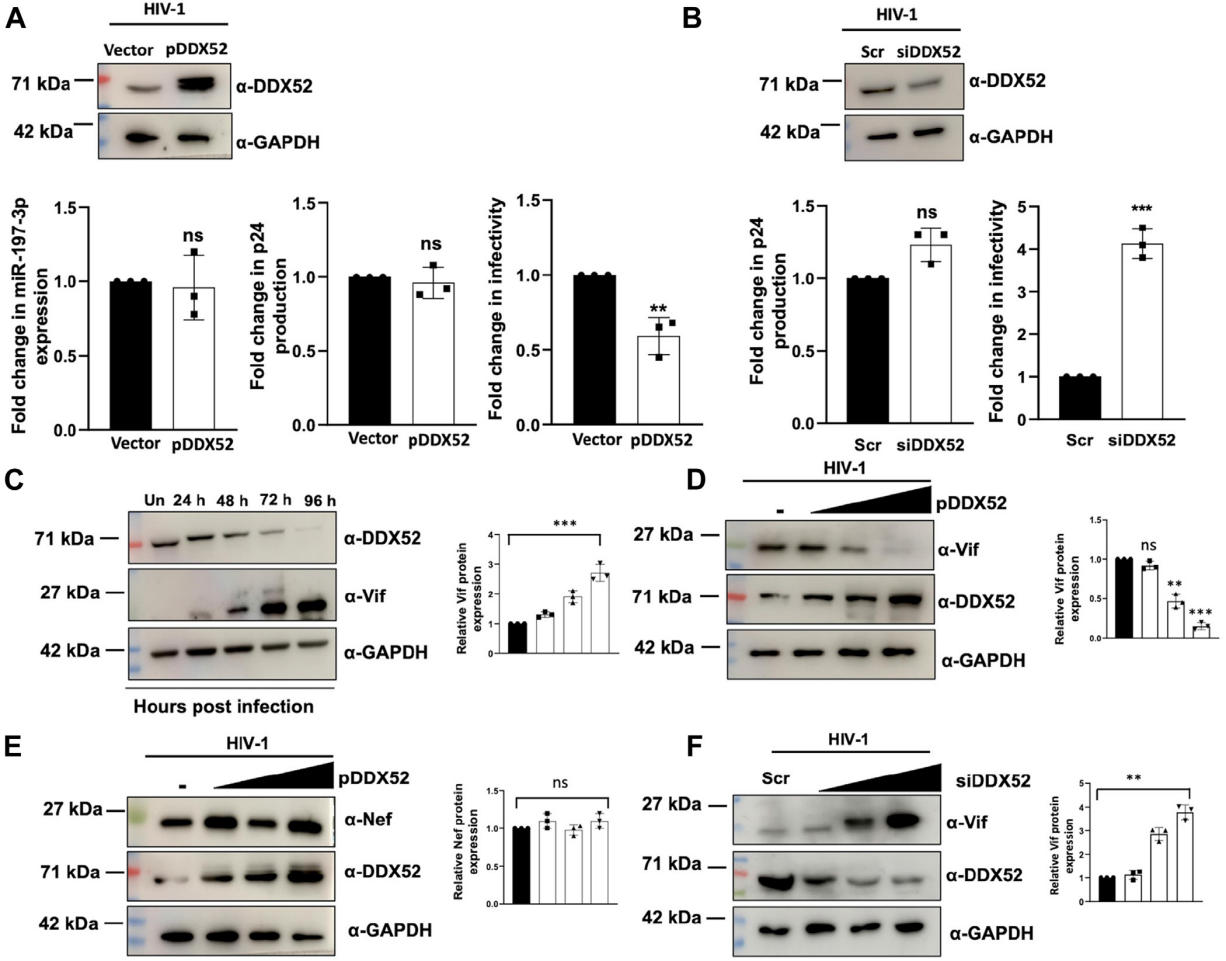
**DDX52 interferes with the infectivity of progeny virions by downregulating cellular expression of HIV-1 Vif.***A*, DDX52 overexpression does not alter the expression of miR-197-3p and virus production but reduces the infectivity of the progeny virions. DDX52 was overexpressed in Jurkat J6 cells, followed by HIV-1 NL4.3 infection (0.5 MOI) 24 h post transfection. The cells were harvested 48hpi and analyzed by immunoblotting and quantitative real-time PCR. Virus production was analyzed by p24 ELISA from the culture supernatants and progeny virion infectivity was compared using TZM-bl reporter cells by X-gal staining. The data were analyzed by two-tailed unpaired Student’s *t* test. *B*, silencing of DDX52 leads to increased progeny virion infectivity, without any effect on virus production. SMARTpool siRNA targeting DDX52 was transfected in Jurkat J6 cells followed by HIV-1 NL4.3 infection (0.5 MOI). p24 ELISA was performed with the supernatants followed by X-gal staining in TZM-bl cells as described in *A*. *Scr*, Scrambled siRNA. The data were analyzed by two-tailed unpaired Student’s *t* test. *C*, expression of DDX52 and Vif proteins show inverse pattern during HIV-1 infection in a T-cell line. HIV-1 NL4.3 (0.5 MOI) infected Jurkat J6 cells were harvested everyday till day 4, and the lysates were used for immunoblotting. *Un*, uninfected. The densitometric analysis is shown on the *right*. *D*, DDX52 overexpression decreases Vif protein expression in a dose-dependent manner during HIV-1 infection. DDX52 was overexpressed in Jurkat J6 cells followed by HIV-1 NL4.3 infection (0.5 MOI). The cells were harvested 48 hpi and analyzed by immunoblotting. The densitometric analysis is shown on the *right*. *E*, DDX52 overexpression has no effect on the expression of Nef. The same lysate from *D* was used to analyze the expression of Nef with the densitometric analysis beside. *F*, siRNA-mediated knockdown of DDX52 increases the expression of Vif. Increasing concentrations of siRNA were transfected followed by HIV-1 infection (0.5 MOI) 24 h post transfection in J6 cells. Cells were harvested 48 hpi and analyzed by immunoblotting. The densitometric analysis is shown on the *right*. The results represent mean ± S.D. from n = 3 independent experiments and statistical significance is defined as ns = *p* ≥ 0.05, ∗ = *p* ≤ 0.05, ∗∗ = *p* ≤ 0.01, ∗∗∗ = *p* ≤ 0.001. Vif, viral infectivity factor; MOI, multiplicity of infection; X-gal, 5-bromo-4-chloro-3-indolyl-β-D-galactopyranoside.

Since DDX52 was downregulated during HIV-1 infection (Fig. 3*E*), we then wanted to determine the outcome of DDX52 overexpression on HIV-1 infection as well as on the expression of miR-197-3p. Jurkat J6 cells were transfected with either empty vector or pDDX52, followed by 0.5 MOI HIV-1 NL4.3 infection after 24 h. It was observed that the expression of the microRNA remains unaltered with DDX52 overexpression when compared with the empty vector transfected HIV-1 infected cells (Fig. 4*A*, left panel). Supernatants collected from the DDX52 overexpressed Jurkat J6 cells showed no significant changes in the virus production (Fig. 4*A*, middle panel). However, the infectivity assay in the TZM-bl indicator cells revealed a significant decrease in progeny virion infectivity (Fig. 4*A*, right panel). Next, the expression of endogenous DDX52 was knocked down in Jurkat J6 cells with the help of a siRNA SMART POOL designed for the gene. Like the overexpression data, there was no significant change in virus production upon silencing which is evident from the p24 ELISA result (Fig. 4*B*, left panel). However, the infectivity assay in TZM-bl cells showed an increase in the infectivity of progeny virions by almost 4-fold on DDX52 knockdown (Fig. 4*B*, right panel) confirming the negative regulatory activity of DDX52 on infectivity. The results thus demonstrate that miR-197-3p and DDX52 have an inverse correlation with respect to modulation of HIV-1 progeny virion infectivity.

The infectivity of the HIV-1 virus has a close relationship with the Vif protein, which governs several important processes like viral RNA folding and packaging processes and is indispensably required in case of infection in the host cells (38). Therefore, it was pertinent to understand the correlation of Vif and DDX52 expression during HIV-1 infection. Our results in Jurkat J6 cells indicated that with infection progression, there was a decrease in the expression of DDX52 with gradual increase in Vif expression (Fig. 4*C*). This suggests that there can be a direct influence of DDX52 expression on the infectivity of the virus through Vif. We then decided to look at Vif expression in correlation with dose-dependent overexpression of DDX52 during HIV-1 infection. The results indicated that with overexpression of DDX52 there was a gradual decrease in the expression of Vif in the infected cells (Fig. 4*D*). Since HIV-1 Nef is also an important viral protein that governs the infectivity of the virus (39, 40), its expression was also examined from the lysates used in the above experiment. However, no significant change in the expression of Nef was observed (Fig. 4*E*), indicating the specificity of DDX52 activity on Vif protein. To further prove the regulatory effect of DDX52 on Vif, DDX52 was silenced with increasing concentrations of siRNA. As expected, the immunoblotting data showed a gradual increase in the levels of Vif upon silencing of DDX52 in HIV-1 infected cells (Fig. 4*F*). Thus, DDX52 seems to be involved in regulation of virion infectivity by modulating Vif protein expression.

### DDX52 downregulates Vif through proteasomal degradation pathway

**Figure 5:**
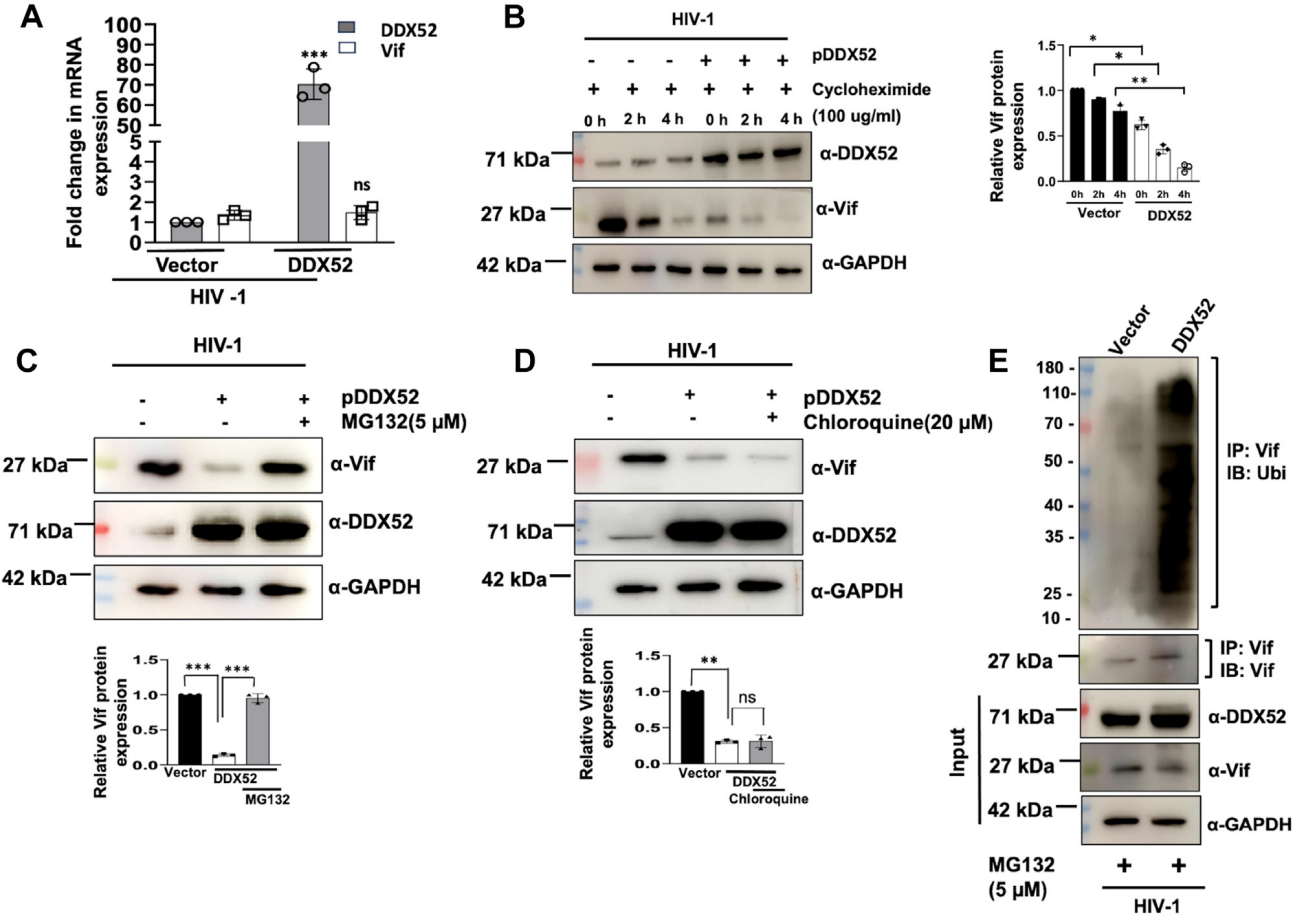
**DDX52 induces proteasomal degradation of Vif.***A*, DDX52 overexpression does not alter Vif mRNA expression during HIV-1 infection. DDX52 was overexpressed in Jurkat J6 cells followed by HIV-1 infection (0.5 MOI) 24 h post transfection. Cells were analyzed for Vif expression at the mRNA level by quantitative real-time PCR 48 h post infection with GAPDH as internal control. The data were analyzed by two-way ANOVA. *B*, DDX52 expression regulates the stability of Vif protein during HIV-1 infection as analyzed from the cycloheximide chase assay. Cycloheximide (100 μg/ml) was added to the cell culture medium for different time duration (0 h, 2 h, and 4 h) prior to the harvesting time point. Lysates were prepared and used for protein expression analysis. Densitometric analysis for the same is shown on the right. *C*, Vif is downregulated *via* proteasomal degradation in the presence of DDX52 as MG132 rescues it. DDX52 was overexpressed in Jurkat J6 cells followed by an infection (0.5 MOI) after 24 h. MG132 was added 12 h before harvesting. Densitometric analysis for the same is shown below. *D*, chloroquine (an autophagy inhibitor) is not able to rescue the degradation of Vif in presence of DDX52. Jurkat J6 cells were overexpressed with DDX52, followed by infection (0.5 MOI) 24 h post transfection and chloroquine was added 12 h before harvesting. Densitometric analysis of the same is shown below. *E*, DDX52 overexpression leads to polyubiquitination of Vif during HIV-1 infection. Jurkat J6 cells were overexpressed with DDX52, followed by HIV-1 infection. MG132 was added to both vector control and DDX52 overexpressed cells 12 h prior to harvesting. The cell lysates were immunoprecipitated with Vif antibody and probed with ubiquitin antibody. The results represent mean ± S.D. from n = 3 independent experiments and statistical significance is defined as ns = *p* ≥ 0.05, ∗ = *p* ≤ 0.05, ∗∗ = *p* ≤ 0.01, ∗∗∗ = *p* ≤ 0.001. Vif, viral infectivity factor; MOI, multiplicity of infection.

Since RNA helicases play a predominant role in various aspects of RNA processing, the downregulation of Vif protein in the presence of DDX52 led us to ask whether DDX52 controls Vif at the transcription level. Interestingly, qRT-PCR analysis showed no change in the expression of *vif* mRNA upon overexpression of DDX52 (Fig. 5*A*) during HIV-1 infection, indicating that DDX52 expression has a negative impact on HIV-1 Vif expression at the protein level alone. We then reviewed the literature to understand the protein level regulation of Vif by DDX52. Studies have shown that DEAD box proteins are involved in a variety of cellular processes, including RNA processing, transcription, translation, RNA transport, and so on. But it is the interaction of a helicase with other proteins present in a multiprotein complex that holds answer for additional functions of these genes, which were previously unknown (41). Hence, their exact functions are largely influenced by their interacting partners and therefore majorly context dependent. To further confirm the protein level regulation of Vif by DDX52, cycloheximide (CHX) assay was performed. Immunoblotting of the protein samples showed a significant and rapid reduction in levels of Vif in presence of DDX52 (Fig. 5*B*) during HIV-1 infection. This indicates that DDX52 overexpression accelerates turnover of Vif indicating clearly that DDX52 controls expression of Vif at the posttranslational level.

The eukaryotic system mostly follows two pathways to degrade target proteins—the proteasome system (responsible for degrading majority of the proteins) and autophagy (mostly involved in the degradation of long-lived and aggregated proteins and also cellular organelles) (42). We then asked whether Vif underwent degradation *via* the proteasomal degradation pathway or the autophagic lysosomal degradation pathway upon ectopic expression of DDX52. To address these possibilities, Jurkat J6 cells were transfected with either an empty vector or DDX52. At 24 h post transfection, the cells were infected with HIV-1 NL4.3 (0.5 MOI). The cells were treated with MG132 at a final concentration of 5 μM 12 h prior to harvesting. Results of immunoblotting showed that DDX52 mediated attenuation of Vif expression was blocked following treatment with MG132 (Fig. 5*C*). In contrast, DDX52-mediated attenuation of Vif expression remains unaffected in chloroquine treated cells (Fig. 5*D*), indicating that DDX52 promotes proteasomal degradation of Vif during HIV-1 infection. Next, to corroborate our findings, we performed ubiquitination assay of Vif in DDX52 overexpressed cells during HIV-1 infection. Jurkat J6 cells were transfected with either empty vector or DDX52. At 24 h post transfection, cells were infected with HIV-1. Cells were treated with MG132 12 h prior to harvesting at 48 h post infection. The lysates were then used for immunoprecipitation using Vif antibody followed by immunoblotting using ubiquitin antibody (Fig. 5*E*). Enhanced Vif ubiquitination indicated by increased laddering in the presence of DDX52 suggests that it regulates Vif expression by inducing proteasomal degradation *via* ubiquitination.

### DDX52 interacts with Vif during HIV-1 infection

**Figure 6:**
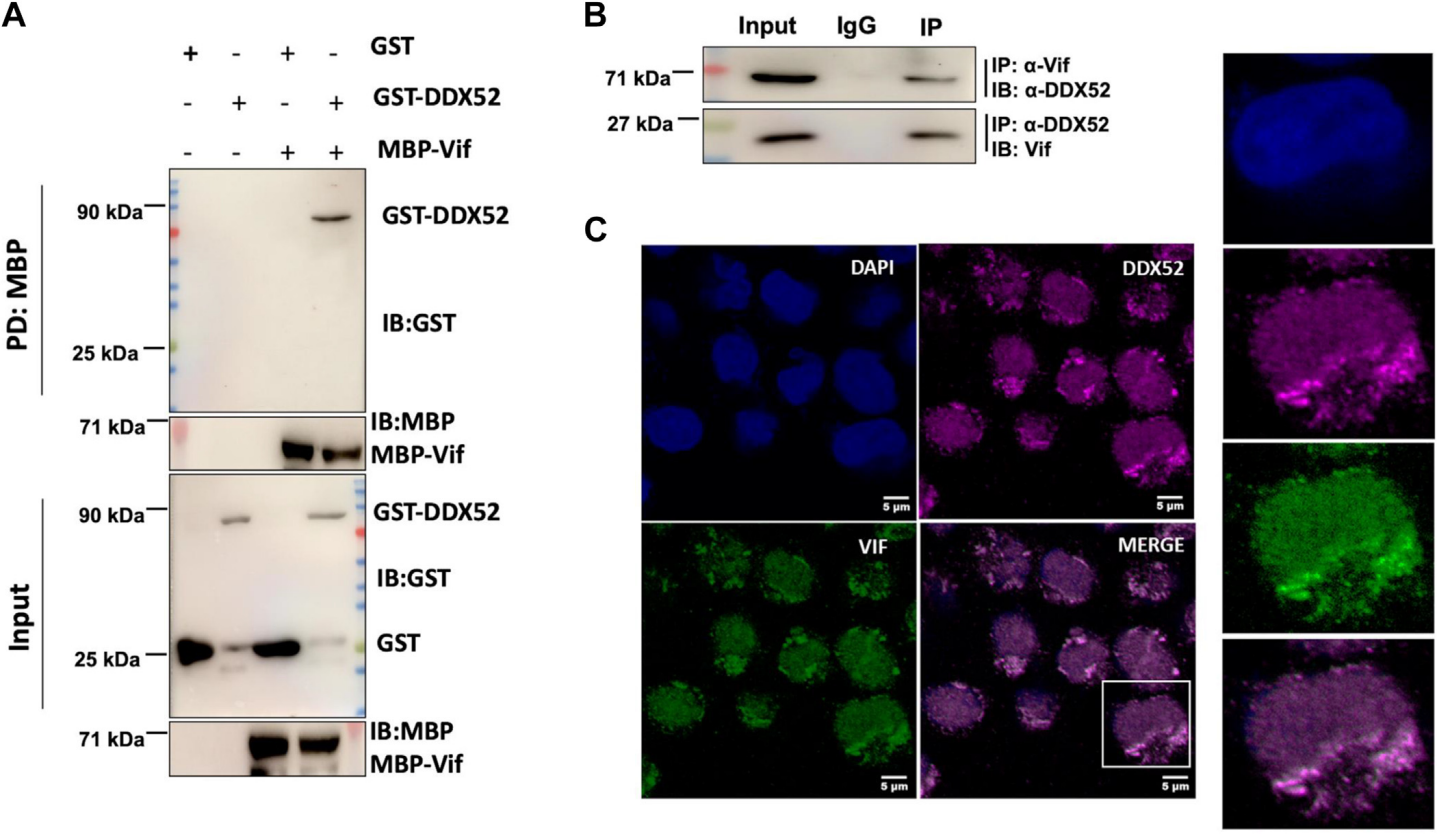
**DDX52 and Vif physically interact with each other during HIV-1 infection.***A*, recombinant purified GST-DDX52 and MBP-Vif directly interact with each other *in vitro*. GST and GST-DDX52 purified proteins were incubated with either MBP affinity resin or affinity resin-bound to MBP-Vif at indicated combinations for 6 h at 4 °C. The MBP affinity resins were then washed and the pulled down proteins were eluted with MBP elution buffer and analyzed by immunoblotting using GST and MBP antibodies. *B*, DDX52 and Vif interact during HIV-1 infection as analyzed by co-immunoprecipitation assay. Jurkat J6 cells were infected with HIV-1 NL4.3 at 0.5 MOI and were harvested at 48 hpi. The cell lysates were analyzed by immunoprecipitation assay with antibodies against DDX52 and Vif. IP, immunoprecipitation; IB, immunoblot. *C*, DDX52 and Vif colocalize and associate in the cytoplasmic compartments of the cell during HIV-1 infection. Jurkat J6 cells were infected with HIV-1 as mentioned in *B*. the cells were harvested 48 hpi and analyzed by confocal microscopy to determine the association of the proteins. The images on the *right* are enlargements of the square in the image on the *left*. The scale bar represents 5 μm. Panels involving A, B, and C are representative of at least three independent experiments. MBP, maltose binding protein; MOI, multiplicity of infection; Vif, viral infectivity factor.

Subsequently, we were interested to examine whether DDX52 physically interacts with Vif during HIV-1 infection. For this, direct interaction studies using recombinant purified proteins, GST-DDX52 and maltose binding protein (MBP)-Vif were first performed. Immunoblotting results demonstrated that DDX52 directly interacts with Vif under *in vitro* conditions (Fig. 6*A*). Additionally, interaction studies between these two proteins in HIV-1 infected cells were also performed. Our results indicated the presence of Vif in co-immunoprecipitation (co-IP) using DDX52 antibody. Similarly, presence of DDX52 was observed with reverse co-IP using Vif antibody (Fig. 6*B*). To determine the subcellular localization of DDX52 and Vif, we then performed the immunofluorescence assay. Jurkat J6 cells, infected with HIV-1 were immunostained for DDX52 and Vif. The confocal microscopy results showed the association of both the proteins in the cytoplasmic compartment of the cells (Fig. 6*C*). These findings indicate that DDX52 interacts with Vif during HIV-1 infection to mediate the downregulation of Vif.

### DDX52 interacts with Vif through the DESD motif that seems to be necessary for downregulation of Vif during HIV-1 infection

**Figure 7:**
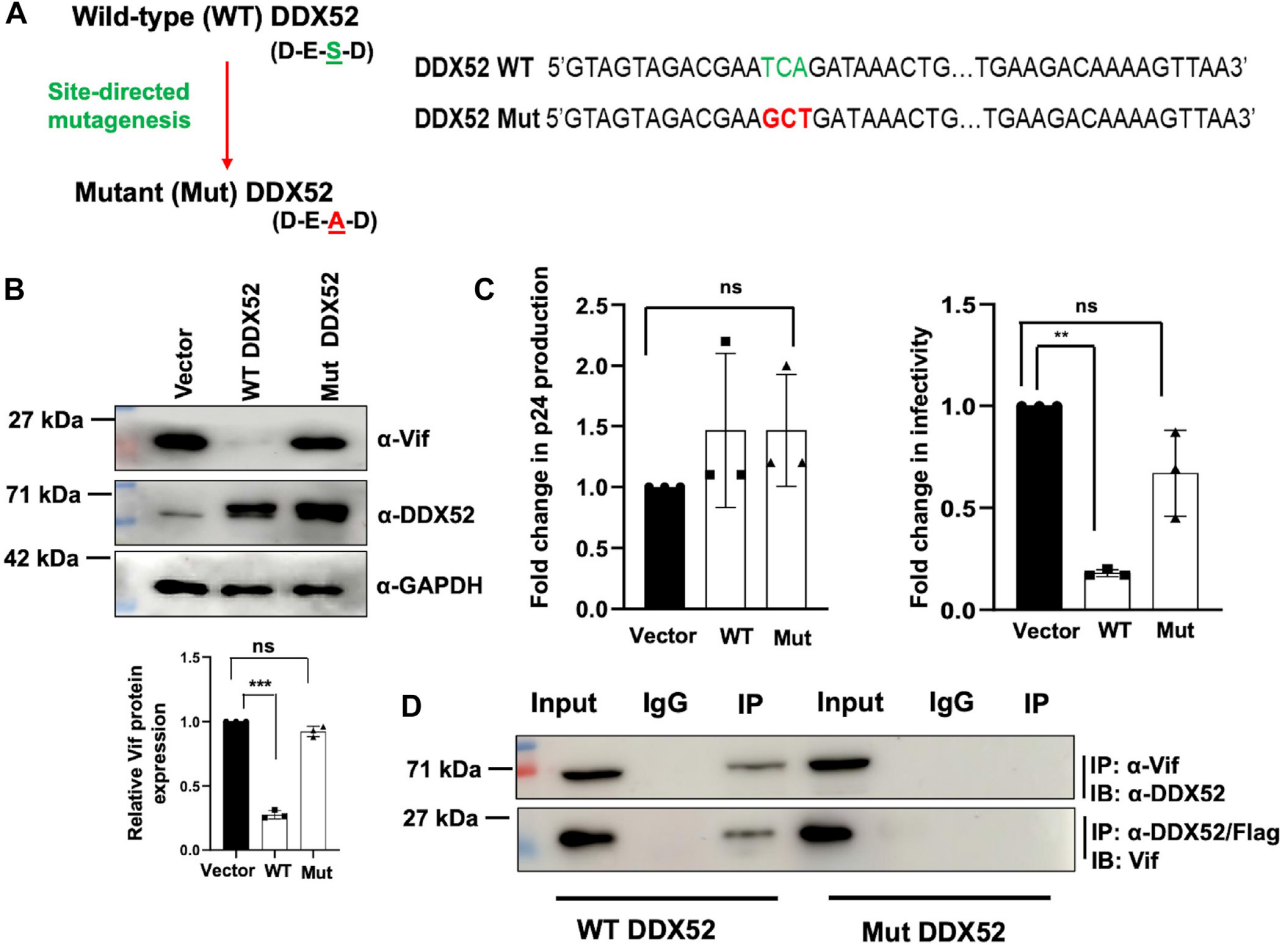
**DDX52 DESD motif is required for interaction with Vif and its downregulation.***A*, schematic representation of the DDX52 sequence to present the position of site-directed mutagenesis from DESD (WT DDX52) to DEAD (Mut DDX52) motif. *B*, Mut DDX52 does not suppress the cellular expression of Vif like the WT DDX52. Jurkat J6 cells were transfected with WT DDX52 and Mut DDX52 (created by site-directed mutagenesis—DESD to DEAD). After 24 h, the cells were infected with HIV-1 NL4.3 at 0.5 MOI. This was followed by harvesting at 48 hpi and immunoblotting of the lysates. *WT*, wild type, *Mut*, mutant. The densitometric analysis is shown below. *C*, expression of Mut DDX52 does not alter the virus production but rescues the infectivity of the progeny virions. The supernatants collected from the above experiment were used to determine the virus production by p24 ELISA (*left* panel) and the infectivity of the progeny virions was determined by TZM-bl infectivity assay (*right* panel) as described earlier. The data were analyzed by one-way ANOVA with Dunnett’s *post hoc* test. *D*, mutant DDX52 fails to interact with Vif during HIV-1 infection as analyzed by immunoprecipitation studies. Similar methodology was followed as discussed in Fig. 6*A*. Panels *A*, *B*, and *C* are representative of at least three independent experiments. The results represent mean ± S.D. from n = 3 independent experiments and statistical significance is defined as ns = *p* ≥ 0.05, ∗ = *p* ≤ 0.05, ∗∗ = *p* ≤ 0.01, ∗∗∗ = *p* ≤ 0.001. Vif, viral infectivity factor; MOI, multiplicity of infection.

According to the literature available till date, most of the host RNA helicases are proviral (43), *i.e.*, the virus uses these RNA helicases for its own benefit. However, the results obtained by us so far suggest that DDX52 might have an antiviral activity. According to the literature, most of the pro-HIV RNA helicases possess the DEAD motif. DDX52 on the contrary, contains the DESD motif. To see if the effect of DDX52 on Vif stability depends on its DESD motif, we created and used a DDX52 mutant (Mut DDX52) containing the DEAD sequence, like the other pro-HIV RNA helicases (Fig. 7*A*). Interestingly, the immunoblotting results demonstrated that, unlike WT DDX52, Mut DDX52 is unable to degrade Vif during HIV-1 infection (Fig. 7*B*). This observation was really intriguing as a change of single amino acid points toward a possible loss of unique functionality of the gene. The supernatants collected from the above experiment were then used to perform p24 ELISA. The results, however, indicated no significant change in virus production, upon either of WT or Mut DDX52 overexpression (Fig. 7*C*, left panel). But the infectivity assay data demonstrated that Mut DDX52 was not able to suppress the progeny virion infectivity (Fig. 7*C*, right panel) unlike the WT DDX52. Moreover, immunoprecipitation assay with the mutant also confirmed the absence of any interaction between Vif and Mut DDX52 (Fig. 7*D*). All these results suggest the importance of the DESD motif of DDX52 for its interaction with Vif as well as its downregulation.

### DDX52 interacts with APOBEC3G in a multi protein complex to regulate the expression of Vif

**Figure 8:**
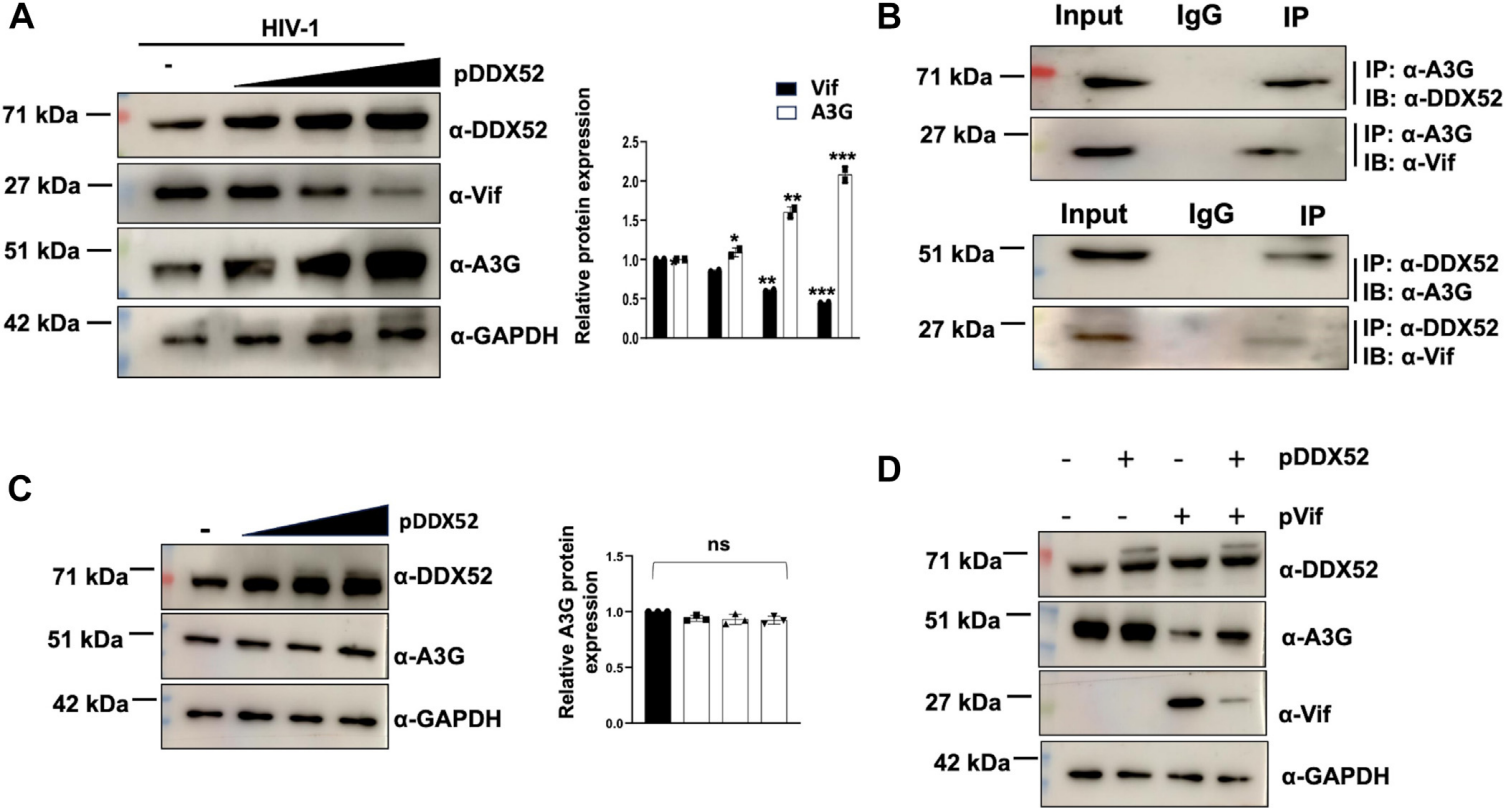
**DDX52 overexpression decreases the expression of Vif and increases the expression of APOBEC3G in a Vif-dependent manner in HIV-1 infected cells and they appear to be part of a multiprotein complex.***A*, DDX52 overexpression leads to increased expression of A3G and downregulation of Vif protein. Jurkat J6 cells were transfected with increasing doses of DDX52 followed by HIV-1 infection (0.5 MOI) 24 h post transfection. The cells were harvested 48 hpi and analyzed by immunoblotting. Densitometric analysis is shown on the *right*. *B*, DDX52 interacts with A3G and Vif in HIV-1 infected cells as analyzed by coimmunoprecipitation assay. Jurkat J6 cells were infected with HIV-1 NL4.3 at 0.5 MOI and harvested 48 hpi. Co-immunoprecipitation assay was performed as described earlier with the indicated antibodies. IP, immunoprecipitation; IB, immunoblot. *C*, DDX52 overexpression has no impact on A3G expression in uninfected Jurkat J6 cells. Uninfected, healthy Jurkat J6 cells were transfected with increasing doses of DDX52. The cells were harvested after 48 h and analyzed by immunoblotting. The densitometric analysis is shown beside. *D*, DDX52 mediated downregulation of Vif rescues A3G expression. Vif was coexpressed with or without DDX52 in uninfected Jurkat J6 cells. The cells were harvested after 48 h and expression analysis of both Vif and APOBEC3G was analyzed by immunoblotting. The upper band visible of DDX52 is the exogenously expressed tagged protein band, while the lower band corresponds to the endogenous expression. Panels *A*, *B*, *C*, and *D* are representative of at least three independent experiments. The results represent mean ± S.D. from n = 3 independent experiments and statistical significance is defined as ns = *p* ≥ 0.05, ∗ = *p* ≤ 0.05, ∗∗ = *p* ≤ 0.01, ∗∗∗ = *p* ≤ 0.001. APOBEC, apolipoprotein B mRNA-editing enzyme, catalytic polypeptide; Vif, viral infectivity factor; A3G, APOBEC3G; MOI, multiplicity of infection.

During its life cycle, HIV-1 interacts with many host proteins which includes the host innate immune response proteins like APOBEC3G (A3G). There is a constant battle that exists between the viral accessory protein Vif and host restriction factor A3G (44, 45) and it is well-known that A3G suppresses Vif expression and *vice versa*. Hence, it was crucial to first determine whether DDX52 expression has any correlation with A3G expression during HIV-1 infection. Immunoblotting data showed a gradual increase in the expression of A3G with concomitant expression of DDX52 (Fig. 8*A*) with a simultaneous decrease in Vif protein expression. This observation advocates toward the possibility of DDX52 having an interaction with A3G to mediate the downregulation of Vif protein. To prove our hypothesis, we performed co-IP assays with HIV-1 infected Jurkat J6 cells. Immunoprecipitations using A3G and/or DDX52 antibodies followed by immunoblotting with respective antibodies demonstrated the interaction of these two proteins during infection (Fig. 8*B*). Since the literature is very clear that both Vif and A3G can cause each other’s degradation *via* the proteasome machinery (29), we also observed the presence of Vif in the same immunoprecipitation samples used above (Fig. 8*B*). To understand if the impact on A3G expression due to DDX52 is indirectly related with the expression of Vif, normal uninfected Jurkat J6 cells were transfected with increasing doses of DDX52. The expression of A3G was then analyzed to verify if DDX52 has any role in it, independent of Vif. We found that dose-dependent increment of DDX52 has no impact on A3G in uninfected cells (Fig. 8*C*). Also, to understand the impact of Vif and DDX52 together on A3G expression, Vif was coexpressed with or without DDX52 in uninfected Jurkat J6 cells, and expression of both Vif and A3G was analyzed. The results clearly indicate that in the presence of Vif, A3G expression is decreased which is rescued to an extent in the presence of overexpressed DDX52 (Fig. 8*D*). These findings collectively indicate that DDX52 mediated downregulation of Vif leads to the rescue of A3G. Moreover, it suggests that the association of DDX52 with A3G is Vif-dependent and these three proteins could be part of a multiprotein complex during HIV-1 infection.

### miR-197-3p inhibition leads to increased expression of DDX52 and APOBEC3G, thereby decreasing progeny virion infectivity

**Figure 9:**
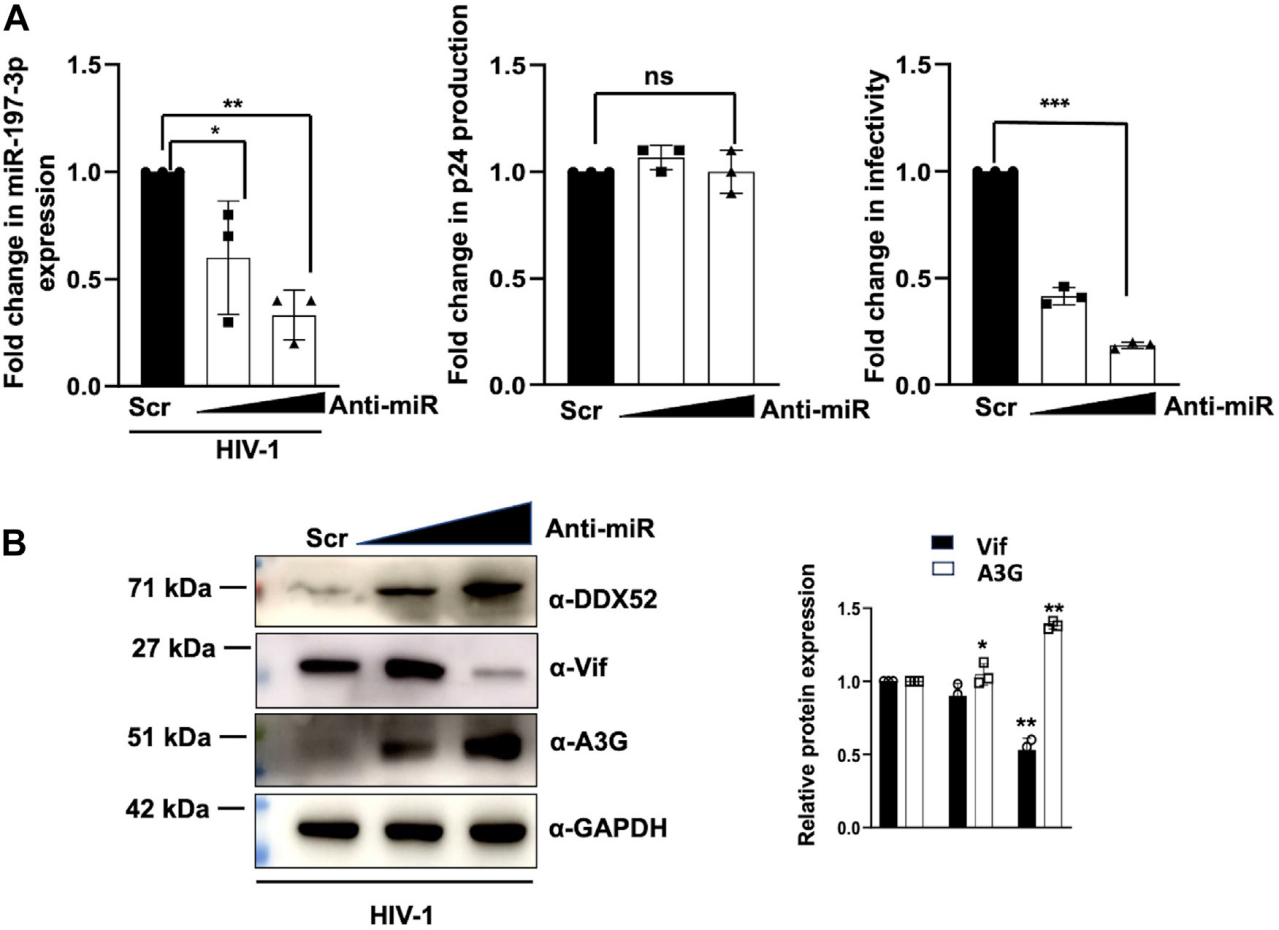
**miR-197-3p knockdown leads to increased expression of DDX52 and APOBEC3G resulting in reduced Vif expression and progeny virion infectivity.***A*, miR-197-3p knockdown leads to a decrease in progeny virion infectivity. Jurkat J6 cells were transfected with anti-miR-197-3p in a dose-dependent manner followed by HIV-1 infection (0.5 MOI) 24 h post transfection. The inhibition of the microRNA expression was confirmed (*left* panel) and after 48 h, the culture supernatants were collected and used for determining virus production by p24 ELISA (*middle* panel). This was followed by TZM-bl *X*-gal staining assay to determine the infectivity of the progeny virions (*right* panel). The data were analyzed by one-way ANOVA with Dunnett’s *post hoc* test. *B*, miR-197-3p knockdown enhances the expression of DDX52 and A3G and decreases the expression of Vif. Jurkat J6 cells were infected with HIV-1 as described above in (*A*), and the cells were analyzed by immunoblotting for DDX52, Vif, and A3G. Densitometric analysis is shown on the *right*. The results represent mean ± S.D. from n = 3 independent experiments and statistical significance is defined as ns = *p* ≥ 0.05, ∗ = *p* ≤ 0.05, ∗∗ = *p* ≤ 0.01, ∗∗∗ = *p* ≤ 0.001. APOBEC, apolipoprotein B mRNA-editing enzyme, catalytic polypeptide; Vif, viral infectivity factor; A3G, APOBEC3G; MOI, multiplicity of infection; X-gal, 5-bromo-4-chloro-3-indolyl-β-D-galactopyranoside.

All our previous results have shown that HIV-1 infection leads to an increased expression of the microRNA miR-197-3p, leading to the downregulation of DDX52 and increased progeny virion infectivity. Hence, we hypothesized that inhibition of the microRNA during HIV-1 infection, might have an opposite impact on the expression of Vif and A3G. To prove this, we transfected Jurkat J6 cells with anti-miR-197-3p in a dose-dependent manner, followed by 0.5 MOI of HIV-1 infection after 24 h. To confirm the downregulation of the microRNA, a qRT-PCR analysis was performed which confirmed the downregulation of the microRNA (Fig. 9*A*, left panel). The supernatants were used for p24 antigen capture ELISA which showed no significant change in the virus production upon inhibition of the microRNA (Fig. 9*A*, middle panel). But interestingly, infectivity assay of these progeny virions in the TZM-bl cells showed a decreased infectivity when the microRNA was silenced (Fig. 9*A*, right panel), thus making it an important player in HIV-1 infection. Since the microRNA was silenced, DDX52 expression was increased, which is evident from the immunoblot data (Fig. 9*B*). When we probed for Vif and A3G proteins in the same samples, we saw that the expression of Vif was reduced complementing the infectivity assay data, while the expression of A3G was increased as expected. Thus, this experimental result clearly suggests that HIV-1 induces miR-197-3p to downregulate DDX52, so that viral infectivity can be increased. Therefore, this study provides another explicit example of how the virus uses host cell machinery like the RNAi pathway, for establishing a successful infection in the host.

## Discussion

HIV-1 has been one of the most serious public health challenges since its discovery in the early 1980s. Although antiretroviral therapeutic regimen is available now that manages the infection satisfactorily, but a complete cure for AIDS is still far from reality along with the issues of drug resistance and latent reservoirs. Therefore, it calls for more in-depth studies of the virus and its interaction with the host cell to understand how the virus uses the host cell machinery.

Noncoding RNAs, especially microRNAs have emerged as very important players in gene regulation in recent times. It is now a proven fact that many microRNAs are differentially expressed under stressful situations like infections and diseases (46, 47). Thus, it becomes important to study the altered expression of microRNAs during HIV-1 infection and their functional implications. Although researchers have identified many microRNAs that are critically involved during HIV-1 infection (14, 16, 19), that number is still small compared to the total number of microRNAs identified in the human genome. Also, several high-throughput studies identifying DEMs during HIV-1 infection have been reported (7, 9, 48), but a comprehensive study to find the common significantly modulated microRNAs in HIV-1 infected human samples has not been performed. Thus, in the present study, we have identified 15 significantly modulated microRNAs from five separate microarray studies performed with HIV-1 infected individuals available in the GEO data base. Among them, miR-197-3p showed consistent and significant upregulation in HIV-1 infected hPBMCs and T-cells.

**Figure 10:**
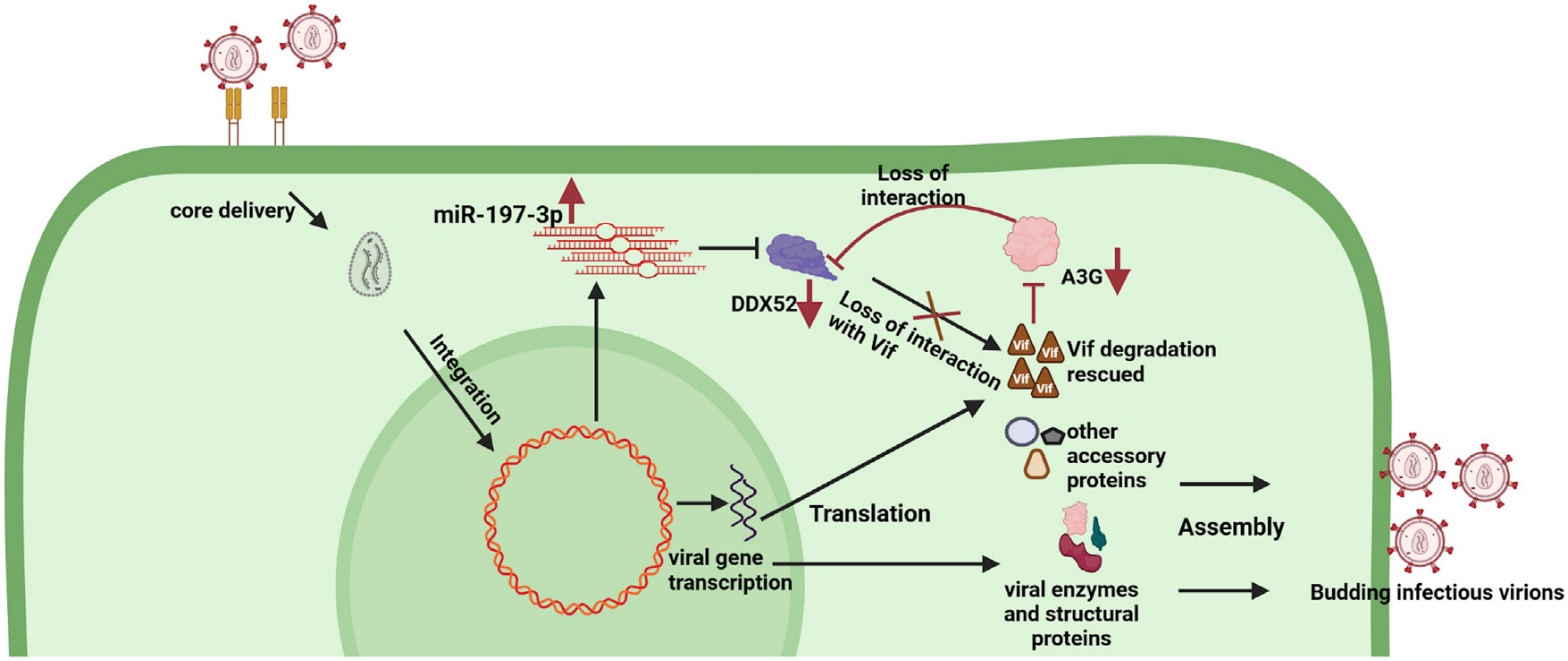
**Schematic representation of how miR-197-3p regulates HIV-1 progeny virion infectivity based on present findings.** During a normal HIV-1 infection, there is enhanced expression of miR-197-3p that leads to suppression of its target DDX52. This increases the expression of Vif protein and thereby it results in budding of infectious progeny virions. (created with BioRender.com). Vif, viral infectivity factor.

Previous studies have shown that miR-197-3p plays a crucial in progression of several cancers like breast cancer (49), bladder cancer (32), hepatocellular carcinoma (33), prostate cancer (50), cervical cancer (51), and so on The mode of action of miR-197-3p reveals that it can play oncogenic function in triple-negative breast cancer and bladder cancer while tumor suppressor roles in cervical, prostate, and hepatocellular carcinoma. However, except prostate cancer, how it functions as tumor suppressor or oncogene is unknown. It was reported that miR-197-3p represses VDAC1/AKT/β-catenin signaling axis to prevent prostate cancer (50). The present study has been done in T-lymphoblast Jurkat J6 cell line (52). However, there are no reports as of yet about miR-197-3p playing any role in T-cell leukemia. Moreover, miR-197-3p is upregulated in HIV-1 infected T cell lines and hPBMCs, indicating that miR-197-3p has a specific positive regulatory function in HIV-1 infection in T cells (Fig. 10). In contrast, miR-197-3p has negative regulatory role only in enterovirus 71 infectious cycle and its expression is diminished following infection (53) indicating that miR-197-3p may function as a positive/negative regulator in pathogen-associated infections in context-dependent manner. Hence, it interested us to decipher its role during HIV-1 infection and delineate its mechanism of action in the present study. Upon both overexpression and silencing of miR-197-3p, we could not find any changes in the virus replication or production, but progeny virion infectivity showed a positive correlation. This was a very exciting finding for us as miR-197-3p might be regulating the infectivity of the virus. We then used bioinformatic target prediction tools to find the target genes of this microRNA and also validated four of the top scoring targets (CD53, DDX52, ETF1, and CLNS1A) *via* reporter-based luciferase assay. Although CD53 gene scored the best among all the targets and was validated as a target of miR-197-3p as well, but we did not study it since detailed functional characterization of CD53 in HIV-1 infection has been already reported (36, 54).

Due to the lack of its own helicase, host RNA helicases such as RNA helicase A and DDX3 are crucial for HIV-1 RNA processing. RNA helicase A was shown to positively control the reverse transcriptase and translational activity of viral RNA (55, 56). Further, it is reported that DDX3 facilitates the function of HIV-1 Tat (57). In our study, we identified RNA helicase DDX52 as a negative regulator of Vif upon HIV-1 infection. Thus, DDX52 functions as a negative regulator of HIV-1 pathogenesis and miR-197-3p functions as a positive regulator of HIV-1 infectivity through attenuating the expression of DDX52. Reports further suggest that RNA helicases have three super families (SF1-3) and two smaller families (F4 and F5) based on distinct motifs and sequence comparisons (58). The most commonly present eukaryotic RNA helicases belong to the SF2 family and they comprise of the DEAD box or the related DExH, DEAH, and DExD motifs in their amino acid sequence (59). DDX52 belongs to SF2 family as it has the DESD motif (DExD motif). We then investigated the impact of DDX52 on its regulator miR-197-3p as well as on virus replication and infectivity. Results showed that overexpression of DDX52 had no significant effect on the microRNA expression. However, expression studies in Jurkat J6 cells suggested that there is an inverse correlation of DDX52 with progeny virion infectivity but without any change in virus production. Although a previous siRNA screening report claimed that upon silencing of DDX52 in HeLa cells, there was a modest decrease in HIV-1 replication but no change in infectivity (60), however, there was a lack of follow-up experiments in the study. In contrast, our results with HIV-1 infected T-cell line Jurkat J6 clearly shows that DDX52 silencing causes no significant change in viral replication but increases HIV-1 progeny virion infectivity. This discrepancy could be due to the use of pseudotyped HIV-1 in HeLa cell system (not a natural host of HIV-1) in the siRNA screening study (60). On the contrary, we have used HIV-1 NL4.3 virus strain in a natural host cell system for our study.

Infectivity of the virus is primarily governed by Vif protein which is a late expressing HIV-1 protein (61). Vif is one of the main weapons of HIV-1 against host cellular restriction factors like A3G as it mediates the latter’s degradation by hijacking the host’s proteasomal degradation system (29, 44, 45). Hence, we decided to look at the regulation of Vif in the presence or absence of DDX52. Dose-dependent silencing and overexpression of DDX52 in HIV-1 infected Jurkat J6 cells showed an inverse correlation with the expression of Vif in these cells. This clearly suggests that DDX52 has a negative impact on progeny virion infectivity, as the expression of Vif is inversely regulated in the presence of host DDX52. It is known that RNA helicases interact with different cofactors to regulate its enzymatic activities as well as subcellular localization to bring about pretranslational and posttranslational modifications of other proteins (62). It is also reported that DEAD box helicases can sense viral RNAs among other aberrations and hence contribute to innate immune signaling (63). In this direction, we wanted to see whether DDX52 regulates Vif expression at the transcription or translation levels. Overexpression studies followed by qRT-PCR analysis showed that there was no change in *vif* expression at the mRNA level, prompting us to move ahead to study protein level regulation. For this, we first looked at the turn-over kinetics of Vif in the presence and absence of DDX52 by the CHX chase assay. We observed a rapid and significant downregulation of Vif suggesting a strict protein level regulation of Vif in the presence of DDX52. Then, we asked if the downregulation of Vif protein was *via* proteasomal or autophagic-lysosomal degradation pathway. We found that addition of MG132 (proteasome inhibitor) rescued the expression of Vif in the DDX52 overexpressed cells. On the other hand, addition of chloroquine (autophagy inhibitor) had no such rescue effect. Our results also suggested that DDX52 and Vif physically interact with each other during HIV-1 infection. Enhanced ubiquitination of Vif in DDX52 overexpressed cells also prompted us to infer that Vif is regulated and degraded in the presence of DDX52 during HIV-1 infection. We were then interested to see if the DESD motif of DDX52 has any role in the regulation of Vif expression. Some reports show that RNA helicases that have the DEAD motif like DDX1, DDX3, DDX5, and DDX21 act in favor of the virus by either helping in the transport of unspliced viral mRNAs from the nucleus to the cytoplasm (64, 65, 66) or by interacting with HIV-1 Tat to enable viral mRNA translation (23). However, in our study we found that the presence of DDX52, which has the DESD motif, tends to lower the infectivity of the progeny virion particles. Therefore, with the help of site-directed mutagenesis, we changed the DESD motif of DDX52 to DEAD motif. It was exciting for us to find that mutant DDX52 expression could no longer cause suppression of Vif. Moreover, mutant DDX52 did not suppress virion infectivity in contrast to the suppression observed with WT DDX52, indicating that DESD motif in DDX52 is important for infectivity of the progeny virion particles. Co-IP assay with the mutant DDX52 also showed absence of interaction with Vif, clearly suggesting the importance of the DESD motif of DDX52 in regulating the expression of Vif and progeny virion infectivity during HIV-1 infection.

In the present study, we found DDX52 to be involved in degrading Vif protein *via* the proteasome degradation machinery. It has been reported earlier that the presence of A3G induces the proteasomal degradation of Vif (29) but there is a lack of clarity in the literature regarding ubiquitination of Vif. So, this intrigued us to ask if there is an association of DDX52 with A3G, the well-known host restriction factor against Vif. We found that dose-dependent increment of DDX52 in HIV-1 infected Jurkat J6 cells increased the expression of A3G, which otherwise remains diminished in the control cells. Also, DDX52 failed to show any impact on A3G in normal uninfected Jurkat cells. It was also observed that DDX52 rescues the expression of A3G attenuated in the presence of Vif. This observation led us to ask if there is an interaction between DDX52 and A3G that prevents the latter from being degraded *via* Vif. It has been shown earlier that MOV10, another host RNA helicase is able to inhibit the proteasomal degradation of A3G mediated by Vif (67). Consequently, the immunoprecipitation assay showed that DDX52 and A3G were interacting partners in HIV-1 infected T cells. Moreover, Vif was also found to be a part of the DDX52-A3G complex. An interaction among these proteins suggests the involvement of a multiprotein complex that causes the degradation of Vif. These findings also provide a possible explanation for the virus induced upregulation of miR-197-3p to inhibit DDX52 from suppressing progeny virion infectivity. While the present study shows a unique association of an RNA helicase with a host restriction factor against HIV-1 Vif, it also opens up new opportunities for understanding the mechanism of proteasomal degradation of Vif in the presence of DDX52 and A3G. For instance, Izumi *et al.* have found that MDM2, which is a known oncoprotein, has the potential to degrade Vif *via* the proteasome machinery during HIV-1 infection (68). Thus, it will be interesting to determine in future studies, whether MDM2 plays a mediating function in Vif degradation *via* any association with A3G and/or DDX52.

In conclusion, our study reveals that microRNA miR-197-3p positively regulates the infectivity of progeny virions of HIV-1 (Fig. 10). It does so through attenuation of RNA helicase DDX52 and A3G to stabilize viral infectivity factor, Vif, at the posttranslational level. Thus, our study reveals a novel miR-197-3p-DDX52-A3G axis to counter HIV-1 infectivity that might be exploited in future for the development of novel antiviral strategy.

## Experimental procedures

### Bioinformatics analysis

#### Microarray data

Differential expression data of microRNAs, generated by microarray studies on HIV-1 infected human samples was obtained from The NCBI GEO (https://www.ncbi.nlm.nih.gov/geo/), a publicly available domain. Studies involving human samples data were included, and cell line data were excluded. The following accession IDs were used for this study: GSE 57323, GSE 33387, GSE 33617, GSE 33837, and GSE 97108 (69, 70, 71). The GSE IDs along with PMIDs, year, platform and the number of HIV-1 infected samples and uninfected controls are shown in Table 1.

### Screening of DEMs in different data sets

For the identification of DEMs, a web-based interactive tool GEO2R (https://www.ncbi.nlm.nih.gov/geo/geo2r/) was used. GEO2R is an user friendly tool that helps to compare two or more groups of samples in a GEO series to identify genes that are differentially expressed across diverse experimental conditions. Differential expression analysis was performed for the normalized expression values for each study mentioned in Table 1. Benjamini and Hochberg FDR method was applied to get the adjusted *p* values corresponding to each microRNA. The multiple *t* test has been applied to screen statistically significant microRNAs after FDR correction. An adj. *p* value < 0.05 and |log FC| ≥1 were set as the primary cutoff criteria for the screening of genes as DEMs. DEMs obtained from all five datasets were intersected using the Venn diagram and the microRNAs that were found to be common for all five datasets were considered for further analysis. The Venn diagram was generated using the Universiteit Gent software (https://bioinformatics.psb.ugent.be/webtools/Venn/). The expression profiles of the selected microRNAs are represented with a heatmap and the heatmap plot was generated using gplots (Gplots. 2016.https://www.rdocumentation.org/packages/gplots/versions/3.1.3), plotly (Plotly. 2017. https://www.rdocumentation.org/packages/plotly/versions/4.7.1) in R-software version 4.2.1 (https://www.R-project.org/).

### Identification of microRNA targets

The potential candidate target genes of the selected DEM were identified by using three well-characterized microRNA target prediction tools available online-miRDB 5.0 (https://mirdb.org/) (72), DIANA tools (http://diana.imis.athena-innovation.gr/DianaTools/index.php?r=MicroT_CDS/index) (73) and TargetScan Human 8.0 (https://www.targetscan.org/vert_80/) (74). These target prediction tools use different algorithms and criteria including base pairing analysis, evolutionary conservation of target sites, accessibility of targets, and so on Predicted list of the target genes with top scores for the selected microRNA was downloaded from each of these databases. To reduce the probability of false-positive read outs, the target genes obtained here were intersected by generating a Venn diagram (https://bioinformatics.psb.ugent.be/webtools/Venn/) and the overlapping genes were then identified for further studies. The target genes were selected based on the scoring criterion for different database and tools (Table 3).

### Cell lines

Buffy coat of anonymous healthy blood donors was procured from the Indian Serological Institute blood bank, Navi Peth, Pune, India. PBMCs were then isolated using Histopaque 1077 (Sigma-Aldrich) by gradient centrifugation as described earlier (75). HEK-293T, a human embryonic kidney cell line, and Jurkat J6, a human CD4+T cell line was obtained from the NCCS Cell Repository, Pune, India. TZM-bl (Cat# 8129) cell line, a derivative of HeLa cells engineered to express CD4, CXCR4, and CCR5 (76) along with stably integrated HIV-1 LTR controlling the reporter genes luciferase and β-galactosidase; and CEM-GFP (Cat# 3655), another CD4+T reporter cell line (77) expressing GFP under the control of HIV-1 LTR was obtained from the NIH AIDS Reagent Program. HEK-293T and TZM-bl cells were maintained in Dulbecco's modified Eagle medium (Invitrogen) while PBMCs, CEM-GFP, and Jurkat cells were grown in RPMI 1640 medium (Invitrogen) supplemented with 10% fetal bovine serum (Invitrogen) and penicillin-streptomycin (Invitrogen) at 37 °C with 5% CO_2_, in a humidified incubator. CEM-GFP cells were also supplemented with 500 μg/ml of G418 (Invitrogen) to maintain the selection pressure.

### Plasmids, vectors, and siRNAs

The HIV-1 molecular clone pNL4.3 (Cat# 114) was obtained from the NIH AIDS Reagent Program, United States (78). pSuper.basic mammalian expression vector (OligoEngine) was a kind gift from Dr Jomon Joseph, NCCS, Pune, and pMIR-REPORT (Thermo Fisher Scientific), which has a luciferase gene downstream to the MCS was a kind gift from Dr Samit Chattopadhyay, NCCS, Pune, India. The sequence of miR-197-3p was cloned in pSuper.basic vector while the WT and mutant 3′UTRs of target genes DDX52, ETF1, and CLNS1A were cloned in pMIR-REPORT mammalian expression vector. DDX52 ORF was cloned in pcDNA6/HisC (Cat# V222–20, Invitrogen) mammalian expression vector and DDX52 mutated ORF was cloned in pcDNA6/HisC and pCMV-Flag2B (Cat# 211172, Agilent technologies) mammalian expression vectors. pGEX-4T1 and pMAL-c2X were used to clone DDX52 and HIV-1 Vif ORF sequences for *in vitro* protein purification. Plasmid pEGFP-N1 (Clontech) was used to normalize transfection efficiency as it encodes GFP under the cytomegalovirus promoter. pCMV-Myc-Vif was kindly given by Dr Akhil C. Banerjea of NII, New Delhi, India (79). anti-miR-197-3p was purchased from Ambion, Life Technologies. Custom Cherry Pick siRNA Library (siGENOME SMART pool) for DDX52 along with nontargeting siRNA was purchased from Dharmacon.

### Antibodies and chemical inhibitors

Primary antibodies against DDX52 (Cat# ab 183848, 1:3000), and HIV-1 Nef (Cat# ab 42358, 1:3000) were obtained from Abcam. Primary antibodies against GAPDH (Cat# sc-32233, 1:4000), HIV-1 Vif (Cat# sc-69732, 1:1000), and GST (Cat# sc-459, 1:50,000) antibodies were obtained from Santa Cruz Biotechnology. Antibody against MBP (Cat# E8032S, 1:4000) was obtained from NEB. APOBEC3G antiserum (Cat# 10082, 1:5000) was procured from the NIH AIDS reagent program, United States (80). Antibody against Ubiquitin (Cat# 13–1600, 1:1000) was obtained from Thermo Fisher Scientific. Antibody against CD53 (Cat# E-AB-62659, 1:1000) was obtained from Elabscience. Flag-tag antibody (Cat# F3165, 1:4000) was obtained from Sigma-Aldrich. Secondary antibodies, goat anti-mouse HRP (Cat# 1706516, 1:4000) and goat anti-rabbit HRP (Cat# 1706515, 1:4000) were procured from Bio-Rad. Cy-3 labeled anti-rabbit immunoglobulin G (IgG) (Cat# AP132C, 1:1000) was obtained from Chemicon, Merck Millipore, and Alexa-fluor 488 anti-mouse IgG (Cat# A-11017, 1:1000) was obtained from Thermo Fisher Scientific. Chemical compounds CHX (Cat# C1988), chloroquine (Cat# C6628), and MG-132 (Cat# 474790) were obtained from Sigma-Aldrich.

### Virus stock preparation

Virus stocks were prepared by transfecting HEK-293T cells with the molecular clone of HIV-1 pNL4.3 using CalPhos mammalian transfection kit (Clontech) as per the manufacturer’s protocol described earlier (81). The cell culture media were collected 24 h post media change, clarified at 1800*g* for 5 min, followed by concentration by ultracentrifugation at 141000*g* in SW28 rotor for 2.5 h at 4 °C in a Beckman Coulter Optima L-90K ultracentrifuge. The viral pellet was then resuspended in serum-free RPMI 1640 with a final concentration of 50 mM Hepes, and small aliquots were made to be stored at −80 °C. The concentration of the virus stock was then calculated using HIV-1 p24 antigen capture ELISA (Advanced Biosciences Laboratories) according to the manufacturer’s protocol.

### HIV-1 infection and quantitation

PHA-activated hPBMCs and/or CD4+ T cell lines (Jurkat J6 and CEM-GFP) were infected with 0.5 MOI HIV-1 NL4.3 virus for 4 h at 37 °C in the presence of polybrene (1 μg/ml) with intermittent mixing. The cells were then washed and resuspended in a complete medium. The hPBMCs were also supplemented with recombinant human IL-2 (Roche Applied Science) at 20 units/ml. The hPBMCs/T cells were then incubated and harvested at 24, 48, 72, and 96 h. The culture supernatants from the infected cells were used to estimate the virus production by p24 antigen capture ELISA, according to the manufacturer’s instructions (Advanced Biosciences Laboratories). The cells were lysed in either TRIzol reagent (Invitrogen) or cell lysis buffer (50 mM Tris–HCl pH 7.4, 5 mM EDTA, 0.12 M NaCl, 0.5 mM NaF, 0.5% NP40, 0.5 mM PMSF, and 1 mM DTT) and used for RNA/protein isolation.

### Viral infectivity assay

To determine the infectivity of the virus generated after HIV-1 pNL4.3 infection or transfection (for virus stock preparation), the culture supernatants were collected and quantified using p24 ELISA. Then, various concentrations of viral p24 units were used to infect TZM-bl reporter cells at a confluency of 50 to 60%. After 48 h, the cells were washed with 1X PBS and fixed with 0.25% glutaraldehyde for 10 min. After washing again with 1X PBS, the cells were stained with X-gal (5-bromo-4-chloro-3-indolyl-β-D-galactopyranoside) as described (75). After 8 to 10 h of incubation, the blue-colored infected cells were counted in at least five random fields, and the average was multiplied with the field factor (depending upon the well plate used) and dilution factor to determine the infectivity of the virus.

### Transient transfection

MicroRNA overexpression in Jurkat J6 cells was achieved by nucleofection with Amaxa Nucleofection kit V (Lonza) using the program X-001. Briefly, three million cells were resuspended in 100 μl of nucleofector solution (Nucleofector solution V + Supplement), and an appropriate amount of expression plasmids was added to the mix. The cell/DNA suspension was then transferred to a certified cuvette such that the sample covers the bottom of the cuvette without any air bubbles. Pulse was given and then 500 μl of complete RPMI was added to the cuvette and transferred to 6 well plates with the final volume made to 1.5 ml. This was followed by HIV-1 infection 24 h post transfection and the cells were harvested 48 h postinfection.

For overexpression studies, HEK-293T and/or Jurkat J6 cells were transfected with indicated expression plasmids using Polyethylenimine, Linear, MW 2500 (Polysciences))/X-tremeGENE HP transfection reagent (Cat# 6366236001) according to the manufacturer’s protocol. Silencing experiments with microRNA inhibitor/gene-specific-siRNA were done using Lipofectamine RNAiMAX (Cat# 13778150, Invitrogen). This was followed by 0.5 MOI HIV-1 NL4.3 infection wherever indicated and the cells were harvested after 48 h. All transfection experiments were normalized using empty vector controls and reporter assays were normalized with EGFP fluorescence readings (82) wherever indicated.

### RNA isolation, reverse transcription, and qRT-PCR

RNA was prepared from infected hPBMCs and Jurkat J6 cells using TRIzol reagent (Invitrogen). For the preparation of complementary DNA for the microRNA of interest, a part of the total RNA was used for polyadenylation and subsequent complementary DNA preparation with the help of miRNA 1st^-^ strand synthesis kit (Agilent technologies), as per manufacturer’s protocol. The expression profile of the selected microRNA was analyzed by qRT-PCR in a 10 μl reaction mixture containing iTaq Universal SYBR Green Supermix (Bio-Rad) and 3.125 μM concentration each of the microRNA’s forward primer, U6 (normalizing control) forward primer and Universal reverse primer (Agilent technologies) using the Realplex^4^ Mastercycler (Eppendorf). The amplification was performed using one cycle of 95 °C for 10 min and 40 cycles of 95 °C for 10 s, 55 °C for 15 s, and 72 °C for 20 s, respectively followed by melt curve analysis. Expression profile of the mRNA of the selected microRNA target genes was also analyzed in a 10 μl reaction mixture containing iTaq Universal SYBR Green Supermix (Bio-Rad) and 10 pmol concentration each of the human GAPDH and target mRNA specific oligonucleotide primer pairs. The amplification was performed using one cycle of 95 °C for 3 min and 40 cycles of 95 °C for 30 s, 60 °C for 30 s, and 72 °C for 30 s, respectively, followed by melt curve analysis. The changes in the threshold cycle (*C*_*T*_) values were calculated by the equation:

The fold difference was calculated as follows:

Fold difference = 2^(−*ΔΔC*^_*T*_^)^

Where, Δ *C*_*T*_ = *C*_*T t*arget_ - *C*_*T*__input_

And ΔΔ *C*_*T*_ = Δ *C*_*T*_ treated - Δ *C*_*T*_ control

The list of all the primers used for qRT-PCR is given in supporting information as Table S1.

### Cloning of miR-197-3p, 3′UTRs of the target genes and DDX52, HIV-1 Vif ORF

miR-197-3p was cloned in pSuper.basic mammalian expression vector. For this, the gene sequence of the microRNA was obtained from the NCBI GenBank (Accession no. NR_029583). The exact sequence of the microRNA was used for synthesizing the forward/sense oligonucleotide strand. The reverse complement sequence of the microRNA was synthesized as the anti-sense oligo strand. The forward strand was given the BglII restriction sequence in the beginning while HindIII restriction site was placed at the end of the reverse sequence. Both these strands were then annealed following the manufacturer’s protocol. This annealed double-stranded sequence was then ligated into the pSuper.basic vector and analyzed for positive clones *via* transformation into *Escherichia coli* DH5α cells. The colonies obtained were selected by restriction enzyme digestion and confirmed by DNA sequencing.

The 3′UTR sequences of the target genes were obtained from the NCBI GenBank (Accession nos. for CD53- NM_001320638, DDX52- NM_007010.5, for ETF1- NM_001291975, for CLNS1A- NM_001293) and oligos were synthesized using specific restriction enzyme sites. After PCR amplification, the products were ligated with the pMIR-REPORT vector that has a luciferase reporter gene upstream of the MCS region. After transformation into *E. coli* DH5α cells, the colonies obtained were screened using restriction enzymes, and the positive clones were confirmed by DNA sequencing.

For cloning of the ORF region of DDX52, the sequence of the ORF region was obtained from the NCBI GenBank (Accession no. NM_007010.5), and oligos were synthesized with specific restriction enzyme sites. After PCR amplification, the product was ligated with the mammalian expression vector pcDNA6/HisC. After transformation, positive clones were selected by restriction enzyme digestion and confirmed by the DNA sequencing. The ORFs of DDX52 and HIV-1 Vif (Accession no. MN685337) were also cloned in pGEX-4T1 and pMAL-c2X protein expression vectors respectively for recombinant protein expression in a similar manner as described above. The list of the cloning primers used here is given in supporting information as Table S2.

### Construction of mutants by site-directed mutagenesis

The clones of the 3′UTR regions of the target genes (CD53, DDX52, ETF1, and CLNS1A) served as the templates for performing site-directed mutagenesis. Here, a single nucleotide alteration in the “seed sequence” of the 3′UTR was done by PCR method. Similarly, a mutant was created in the DESD motif of DDX52 following the same method as described above, where the DDX52 ORF clone was used as the template. Positive clones were chosen using restriction digestion and later confirmed by DNA sequencing. The list of the primers used here is given in supporting information as Table S2.

### Immunoblotting

For cell lysis, buffer containing 50 mM Tris–HCl pH 7.4, 5 mM EDTA, 0.12 M NaCl, 0.5 mM NaF, 0.5% NP40, 0.5 mM PMSF, and 1 mM DTT, supplemented with Protease Inhibitor cocktail (Roche Applied Science) was added to the cells and kept on ice for 45 min with intermittent vortexing. Protein concentration was quantified using Bradford assay reagent (Bio-rad). Equal amounts of protein were then loaded on a 10 to 12% SDS-PAGE gel, followed by transfer to a polyvinylidene fluoride membrane (Millipore). Blocking was done in 5% skimmed milk or bovine serum albumin and then probed with respective antibodies. After the primary and secondary antibody incubation, the blots were developed using Clarity or Clarity-max Western ECL substrate (Bio-rad). For the immunoblots, all densitometric analyses were performed using ImageJ software 1.8.0_345 [64 bit] by normalizing to the respective GAPDH levels (https://imagej.net).

### Recombinant protein isolation

Recombinant proteins (GST/GST-DDX52, MBP/MBP-Vif) used in the study were purified using *E. coli* BL21 bacterial system as described previously (83). Briefly, the BL21 bacterial cells were transformed with the desired recombinant plasmids. To express the proteins of interest, a single colony was inoculated and was allowed to grow at 37 °C overnight with constant agitation under antibiotic selection. The next day, a secondary culture was inoculated from the overnight grown primary culture at 37 °C until the cells reached the early log phase of growth (*A*_600_ ∼ 0.6–0.8). The cells were then induced with 0.5 mM IPTG and were allowed to grow further at 18 °C for 14 h. The cell pellets were resuspended in lysis buffer (50 mM Tris—HCl, 500 mM NaCl, 1 mM DTT pH 7.4, and 1 mM PMSF) containing 0.4 mg/ml lysozyme and sonicated for six cycles with a 15 s pulse (15 s ON and 15 s OFF) at an amplitude of 80%. The cell lysates were then collected and incubated with either Glutathione agarose beads (Cat# 16101, Thermo Fisher Scientific) or MBP affinity resin (Cat # AB270538 Abcam). The recombinant proteins were then eluted with either 3 mg/ml reduced glutathione (pH 8.0) for GST and GST-DDX52 or MBP elution buffer (50 mM maltose, 20 mM Tris–HCl, 200 mM NaCl, 1 mM EDTA, and 1 mM DTT, pH 7.4). Protein concentration was quantified using Bradford assay reagent (Bio-rad).

### *In vitro* pull-down assay

Protein-protein interaction between recombinant DDX52 and Vif was determined using *in vitro* pull-down assay as described previously (83). Concisely, recombinant MBP-Vif was incubated with purified GST or GST-DDX52 for 6 h at 4 °C in combinations as indicated. As control, MBP beads were also incubated with either purified GST or GST-DDX52. The beads were then washed thrice with 0.1% NP40 in 1X PBS and eluted with MBP elution buffer. The samples were then boiled in 2X Laemmli buffer and resolved in SDS-PAGE, followed by Western blotting with the indicated antibodies.

### Co-immunoprecipitation

For the co-IP assays, Jurkat J6 cells were infected with 0.5 MOI HIV-1 NL4.3. After 48 h, the cells were harvested and lysed in cell lysis buffer. Then the lysates were precleared with the indicated IgG antibodies for 1 h at 4 °C. This was followed by primary antibody incubation overnight at 4 °C. The next day, Agarose A/G beads (Invitrogen) were added to the antigen-antibody complex and again incubated for 4 h at 4 °C. The complexes were then pulled down gently by centrifugation and the pellets were washed thrice with 0.1% NP40 in 1X PBS. After this, the samples were boiled in 2X Laemmli buffer (4% SDS, 20% glycerol, 10% 2-mercaptoethanol, 0.004% bromophenol blue, and 0.125 M Tris–HCl, pH 6.8) and resolved on a 10% SDS gel. Proteins were transferred to the polyvinylidene fluoride membrane and probed with the indicated antibodies. The blots were developed using Clarity or Clarity-max Western ECL substrate (Bio-rad).

### Immunofluorescence

For immunofluorescence studies, Jurkat J6 cells were infected with 0.5 MOI HIV-1 NL4.3 and the cells were harvested after 48 h. After washing twice with 1X PBS, the cells were fixed with 4% paraformaldehyde in 1X PBS for 20 min at room temperature. After that, paraformaldehyde was removed, and the cells were incubated with 0.1% glycine for 5 min. Cells were then washed twice with 1X PBS and attached to the slides by cytocentrifuge. Next, the cells were permeabilized with 0.1% Triton X-100 for 10 min, followed by washing twice with 1X PBS. Blocking was done in 4% bovine serum albumin for 2 h at room temperature, followed by washing with 1X PBS. The slides were then incubated with primary antibodies (α-DDX52 and α-Vif) overnight at 4 °C. The next day, cells were washed thrice with 1X PBS, followed by secondary antibody incubation- α-rabbit-Cy3-labeled and α-mouse-Alexa Fluor 488-labeled antibodies respectively, for 2 h. The cells were washed thrice and mounted using mounting media along with 4′,6-diamidino-2-phenylindole (Cat# sc-24941, Santa Cruz Biotechnology). Immunofluorescence was acquired on an Olympus FV3000 Confocal Microscope.

### CHX chase assay

The CHX chase experiment was used to determine the turnover kinetics of Vif protein in the presence or absence of DDX52. For this, Jurkat J6 cells were transfected with pcDNA6/HisC empty vector or the construct of DDX52, followed by infection with HIV-1 NL4.3 (0.5 MOI) 24 h post transfection. After 2 days, prior to 4 h of harvesting, the cells were treated with CHX (100 μg/ml). Immediately after addition, some cells were harvested and they were designated as 0 h. The next batch of cells were harvested after 2 h of drug addition (2 h), and the final harvest was done after 4 h, which was then followed by protein extraction and expression analysis *via* immunoblotting.

### Statistical analysis

All the experiments were replicated at least thrice. The graphs were plotted and analyzed using GraphPad Prism 8.00 Version (https://www.graphpad.com, GraphPad Software, Inc). The statistical analysis of the experimental data were done with unpaired 2-tailed Student’s *t* test for comparison between two groups of data and one-way/two-way ANOVA for more than two groups of data as and when necessitated. The results are plotted as mean ± standard deviation (SD). The significance level is shown in the figures as ns = *p* ≥ 0.05, ∗ = *p* ≤ 0.05, ∗∗ = *p* ≤ 0.01, ∗∗∗ = *p* ≤ 0.001.

## Data availability

The data that support the findings of this study are available in this article. Further details of data and materials will be made available upon reasonable request to the corresponding author.

## Supporting information

This article contains supporting information.

## Conflict of interest

The authors declare that they have no conflicts of interest with the contents of this article.
